# Whole genome sequencing completes the molecular genetic testing workflow of patients with Lynch syndrome

**DOI:** 10.1038/s41525-025-00461-z

**Published:** 2025-01-18

**Authors:** Klaudia Horti-Oravecz, Anikó Bozsik, Tímea Pócza, Ildikó Vereczkey, Tamás Strausz, Erika Tóth, Tatiana Sedlackova, Diana Rusnakova, Tomas Szemes, István Likó, Edit Oláh, Henriett Butz, Attila Patócs, János Papp, Vince Kornél Grolmusz

**Affiliations:** 1 Department of Molecular Genetics, National Institute of Oncology, Budapest, Hungary; 2 Semmelweis University Doctoral School, Budapest, Hungary; 3 National Tumorbiology Laboratory, National Institute of Oncology, Budapest, Hungary; 4 Hereditary Tumors Research Group, HUN-REN – Semmelweis University, Budapest, Hungary; 5 Department of Surgical and Molecular Pathology, National Institute of Oncology, Budapest, Hungary; 6 Comenius University Science Park, Bratislava, Slovakia; 7 Geneton Ltd., Bratislava, Slovakia; 8 Department of Molecular Biology, Faculty of Natural Sciences, Comenius University, Bratislava, Slovakia; 9 Department of Laboratory Medicine, Semmelweis University, Budapest, Hungary; 10 Department of Oncology Biobank, National Institute of Oncology, Budapest, Hungary

**Keywords:** Cancer genetics, Risk factors

## Abstract

Multigene panel tests (MGPTs) revolutionized the diagnosis of Lynch syndrome (LS), however noncoding pathogenic variants (PVs) can only be detected by complementary methods including whole genome sequencing (WGS). Here we present a DNA-, RNA- and tumor tissue-based WGS prioritization workflow for patients with a suspicion of LS where MGPT detected no LS-related PV. Among the 100 enrolled patients, MGPT detected 28 simple PVs and an additional 3 complex PVs. Among the 69 MGPT-negative patients, the lack of somatic *MLH1* promoter methylation in a patient with a distinguished *MLH1* allelic imbalance selected this sample for WGS. This returned a germline deep intronic *MLH1* variant, with further functional studies confirming its’ pathogenicity. Interestingly, all three complex PVs and the *MLH1* deep intronic PV were found to be recurrent at our center. Our straightforward and cost-effective prioritization workflow can optimally include WGS in the genetic diagnosis of LS.

## Background

Lynch syndrome (LS) originally described by Warthin^1^ is a frequent cancer predisposition condition resulting from germline pathogenic variants in *MLH1*, *MSH2*, *MSH6*, *PMS2* and *EPCAM* genes affecting DNA mismatch repair. The growing availability of next-generation sequencing-based multigene panel testing (MGPT) revolutionized the genetic diagnosis of families with LS. In addition to single nucleotide variants (SNV), simple copy number variations including larger deletions and duplications (CNVs) and complex structural variations (SVs) might also serve as pathogenic variants (PVs) resulting in LS. In the case of *MSH2* and *PMS2*, 20–25% of all pathogenic variants are CNVs and SVs, while these are less frequent in *MLH1* ( ~10%) and *MSH6* ( ~3%)^2^. Nevertheless, our research group has previously shown that germline 3’ deletions of *EPCAM* (formerly known as *TACSTD1*) are additional PVs in LS^3^.

Although MGPT has a high yield in detecting SNVs and CNVs, the validation of complex SVs is more challenging and often requires the multimodal application of several molecular genetic techniques and bioinformatic pipelines. The precise characterization of complex variants found in LS is necessary to enhance adequate nomenclature and to determine transcriptional relevance and pathogenicity. The confirmation of altered splicing or premature stop codon is necessary for optimal clinical interpretation of such variants as PVs.

Nevertheless, MGPTs cannot identify PVs located outside of the targeted exons, which usually account for the missing heritability in LS^4^. The detection of these variants requires the systematic analysis of the non-coding genome by targeted deep-intronic and long-read sequencing or whole genome sequencing (WGS) as confirmed in case series of families with high cancer predisposition, which were collected throughout variable time frames^4–6^. These studies clearly showed the robustness of the whole-gene/whole-genome-based approaches, however, a clear workflow regarding the precise role of these tools in the clinical genetic diagnostic setting is lacking.

Here, we present a comprehensive molecular genetic workflow, prospectively applying germline DNA, RNA and tumor tissue-based techniques for the diagnosis of LS. In a pilot study of 100 consecutive patients with colorectal or endometrial cancers fulfilling the testing criteria, MGPT detected 31 PVs, among which 3 were complex PVs, which were subjected to further characterization. In cases where MGPT failed to detect a PV, tumor tissue-based methylation assay targeting the *MLH1* promoter as well as RNA-based analysis of allelic imbalance were conducted to select high-risk samples for WGS. In the sole sample from this cohort selected for further testing, WGS detected a deep intronic PV in *MLH1* causing exonization. Interestingly, this novel variant and all of the detected complex PVs were found to be recurrent in apparently independent families diagnosed at our center, highlighting their relative importance in this population. Our results present a straightforward clinical laboratory genetic workflow leveraging multiple assays for the optimal diagnosis of LS.

## Methods

### Patients and samples

One hundred individuals from 99 independent families diagnosed with colorectal (CRC) or endometrial (EC) cancers and fulfilling testing criteria for LS were consecutively enrolled at the Department of Molecular Genetics, National Institute of Oncology between September 2021 and March 2023. Testing criteria adhered to valid National Comprehensive Cancer Network (NCCN) and Hungarian national guidelines and the modified Bethesda criteria^7,8^. Patients with mismatch-repair deficient/microsatellite instable cancers or conforming the modified Bethesda criteria were included^7^. Patients underwent genetic counseling and provided written informed consent. DNA was isolated from peripheral blood using Gentra DNA Blood Extraction Kit (Qiagen, Hilden, Germany) according to the manufacturer’s instructions. The study was approved by the Scientific and Research Ethics Committee of the Medical Research Council of Hungary (ETT-TUKEB 53720-7/2019/EÜIG) and complied with national and international regulations including the Declaration of Helsinki. Genetic counseling was provided to all participants. All participants provided written informed consent. Family trees were edited using f-tree software (v4.0.3, https://www.holonic-systems.com/f-tree/en/).

### Immunohistochemical evaluation of tumors

Immunohistochemical analyses were performed at the Department of Surgical and Molecular Pathology of the National Institute of Oncology. Briefly, 2-3 µm-thick formalin-fixed paraffin-embedded tissue sections were transferred to a 50 °C water bath (Tissue Flotation Bath TFB 45, Medite) and mounted on a glass slide, which underwent incubation for 1 hour in a drying oven at a temperature of 55 °C and were stored at 4 °C until staining. The BenchMark ULTRA system (Roche Tissue Diagnostics) was applied for fully automated immunohistochemical staining. In the case of MLH1, MSH2 and MSH6 ultraView DAB (Ventana Medical Systems, Cat. No.: 760-500, v.1.02.0018) method was used. After slide heating and deparaffinization at 72 °C for 4 minutes, cell conditioning was performed with ULTRA Cell Conditioning Solution (ULTRA CC1, Ventana Medical Systems, Cat. No.: 950-224) for 3 hours at 95 °C. Slides were incubated with one drop of anti-MLH1 (M1) mouse monoclonal antibody (1 μg/mL, Ventana Medial Systems, Cat. No.: 790-5091), anti-MSH2 (G219-1129) mouse monoclonal antibody (20 μg/mL, Ventana Medial Systems, Cat. No.: 790-5093) or anti-MSH6 (SP93) rabbit monoclonal antibody (1 μg/mL, Ventana Medial Systems, Cat. No.: 790-5092) with cover oil for 16 minutes. For background staining one drop of Hematoxylin II (Ventana Medial Systems, Cat. No.: 790-2208) with cover oil was applied for 12 minutes. One drop post-background stain using bluing reagent (Ventana Medial Systems, Cat. No.: 760-2037) with cover oil was applied for 12 minutes.

In the case of PMS2 U OptiView DAB IHC (Ventana Medical Systems, Cat. No.: 760-700, v.1.00.01.36) method was used. After slide heating and deparaffinization at 72 °C for 4 minutes, cell conditioning was performed with ULTRA CC1 for 580 minutes at 100 °C. After peroxidase inhibition with a 3.0% hydrogen peroxide solution, slides were incubated with one drop of anti-PMS2 (A16-4) mouse monoclonal antibody (1 μg/mL, Ventana Medial Systems, Cat. No.: 790-5094) for 32 min. One drop 0.04% hydrogen peroxide in phosphate buffer solution OptiView H_2_O_2_ was used for 4 minutes. OptiView HRP Multimer containing a 40 μg/mL monoclonal anti-HQ-labeled HRP tertiary mouse antibody in a buffer containing protein and 0.05% ProClin 300 preservative with cover oil was incubated for 4 minutes. For background staining one drop of Hematoxylin II with cover oil was applied for 12 min. One drop post-background stain of bluing reagent with cover oil was applied for 12 minutes.

### Multigene panel testing (MGPT) and variant interpretation

Next-generation sequencing library preparation has been performed using the TruSight Hereditary Cancer Panel and sequencing was run on a MiSeq or NextSeq550DX instruments with appropriate reagent kits (Illumina Inc, San Diego, CA, USA). Genomic alignment and variant calling including SNVs, CNVs and SVs were performed in the DRAGEN Enrichment (version 4.2.4, Illumina Inc, San Diego, CA, USA). Variants correspond to the MANE Select transcript (version 1.3) of each gene (*MLH1*: NM_000249.4; *MSH2*: NM_000251.3; *MSH6*: NM_000179.3; *PMS2*: NM_000535.7; *EPCAM*: NM_002354.3). Clinical variant interpretation was performed according to the ACMG guidelines^9,10^ and variants were cross-checked in the InSight (http://insight-database.org/) and ClinVar (https://www.ncbi.nlm.nih.gov/clinvar/) databases (finalized October 31, 2023). All pathogenic and likely pathogenic variants were validated from an independent blood sample using Sanger sequencing (SNVs) or multiplex ligation-dependent probe amplification (MLPA in the case of CNVs). In the case of validating *PMS2* SNVs, long-range PCR was used to specifically exclude amplification from the pseudogene.

### Somatic *MLH1* promoter methylation analysis

DNA was isolated from formalin-fixed paraffin-embedded CRC and EC tissues at the Department of Surgical and Molecular Pathology of the National Institute of Oncology by the Maxwell® RSC DNA FFPE Kit (Promega, Cat. No.: ASB1450), following the manufacturer’s recommendations. DNA samples were bisulfite converted using the EZ DNA Methylation‐Lightning kit (Zymo Research, Cat. No.: D5031) according to the manufacturer’s instructions. The *MLH1* promoter region was amplified by touchdown PCR reaction (14 cycles with gradually decreasing annealing temperatures from 62°C to 55°C, followed by 26 cycles with annealing temperature of 55 °C^11^, Table S1A). Sanger sequencing was performed on an Applied Biosystems 3500 Genetic Analyzer (ThermoFisher Scentific, MA, USA).

### Peripheral blood mononuclear cell isolation

Peripheral blood mononuclear cells (PBMCs) were isolated by density gradient centrifugation, as earlier reported^12^. Briefly, 9 ml whole blood was layered on 5 ml Pancoll solution (PanBiotech, Cat. No.: P04-601000) and was centrifuged (800 g, 20 min). PBMCs were washed twice with PBS (Corning, Cat. No.: 21-040-CV) and cryopreserved in a 90% FBS-10% DMSO solution (PanBiotech, Cat. No.: P60-36720100) in liquid nitrogen.

### RNA isolation and cDNA synthesis

RNA was isolated (Qiagen, Cat. No.: 217004) from PBMC samples, according to the manufacturer’s instruction after centrifugation with QIAshredder (Qiagen, Cat. No.: 79654). Following RNA extraction, concentration was determined by Nanodrop (Cat.No.: ND-1000). Reverse transcription was performed by the application of ProtoScript® II First Strand cDNA Synthesis Kit (New England Biolabs, Ipswich, MA, USA).

### Analysis of allelic imbalance

In patients selected for allelic imbalance analysis, RT-PCR amplification was performed on the cDNA and positions of germline transcribed heterozygote variants were assessed using Sanger sequencing of both gDNA and cDNA (Table S1B). The presence of a more than 50% decrease in electrophoretic peak intensity of either allele in comparison to the gDNA-based result was considered an allelic imbalance.

### Nonsense-mediated mRNA decay (NMD) inhibition

NMD inhibition has been performed as described previously, with minor modifications^13,14^. Briefly, cells were thawed rapidly in a 37 °C water bath, centrifuged for 10 min at 300 g and underwent incubation for 120 hours in 10 ml PB-MAX Karyotyping Medium (Gibco, Cat. No.: 12557-021) at 37 °C in 5% CO_2_ atmosphere. Thereafter, cells were treated with 200 μL 10 mM (end concentration 200 μM) puromycin (Merck, Cat. No.: P4512) for 6 h at 37 °C to achieve translation inhibition. The same volume of vehicle (distilled water) was added to the negative controls. Following puromycin treatment, cells were harvested, centrifuged at 250 g for 5 min, resuspended in QIAzol (Qiagen) and stored at -80 °C until RNA isolation.

### Whole genome sequencing

Whole genome libraries were prepared using the Illumina DNA PCR-Free Prep, Tagmentation kit (Illumina, USA), with a standard input of 400 ng of gDNA as described in the manufacturer’s guide. To remove free adapters from the final libraries, a cleaning step with AMPure XP magnetic beads (Beckman Coulter, USA) at a ratio of 1:1 was added. The concentration of WGS libraries was measured using the Qubit ssDNA Kit (Thermo Fisher Scientific, USA). The quality of next-generation sequencing (NGS) libraries was assessed using automated electrophoresis on a 2100 Bioanalyzer System (Agilent, USA). For normalization of prepared NGS to 2 nM, a library length of 450 bp was used. The generated libraries were further diluted and denatured according to the NovaSeq 6000 Denature and Dilute Libraries Guide (Illumina, USA), and sequenced using the S4 Reagent Kit v1.5 (300 cycles) (Illumina, USA) with the Standard Loading workflow. A sequencing run with a 2 × 151 cycles configuration was performed on the NovaSeq 6000 instrument (Illumina, USA).

Sequencing files in BCL format were converted into demultiplexed FASTQ-format files using bcl2fastq v2.20.0.422 with default settings and one barcode mismatch allowed. Quality control of the FASTQ files was done in FastQC. FASTQ files were further processed in the DRAGEN environment (Version 4.2.4). Illumina DRAGEN Multigenome Graph Reference hg38 (alt-masked_graph+cnv+hla+rna_v3) was used as the reference genome. VCF files were generated for SNV, CNV and SV and were filtered using Basespace Variant Interpreter (v2.17.0.60) with >10 Total Read Depth, >5 Alt Allele Depth, >0.2 variant read frequency. VCF sequence variation descriptions were converted into the HGVS format with Variant Validator (https://www.variantvalidator.org/service/validate/batch/). Variant call annotation was performed using the UCSC Variant Annotation Integrator (https://genome.ucsc.edu/cgi-bin/hgVai) using the MANE transcript and all available annotations. Additional variant annotation including splice effect predictions were performed in Ensembl Variant Effect Predictor (https://www.ensembl.org/Homo_sapiens/Tools/VEP/), where masked and raw SpliceAI, MaxEntScan, dbscSNV ADA and RF scores were applied. Further splice annotation was performed with CI-spliceAI (https://ci-spliceai.com/). Population-level frequencies of analyzed variants were queried from the Genome Aggregation Database (gnomAD Genomes 3.1.2 v2).

### Molecular genetic characterization of complex PVs

Complex SVs underwent a detailed characterization including the application of MLPA and cDNA-based assays to determine the genomic architecture and transcriptional relevance, respectively. In the case of the *MLH1* SV detected in patient #10, cDNA was amplified MLH1_C6-F and MLH1_C7-R (Table S1B) and the SV-specific PCR detecting the breakpoint was performed with primer pairs MLH1_int18_tp_AS and MLH1_int18_tp_S (Table S1C). Regarding the cDNA analysis of the MSH2 SV detected in patient #97, primer pairs MSH2_C7-F and MSH2_C7-R were used (Tables S1B and S1D).

Allelic imbalance regarding the *MLH1* c.93 G > A variant in patient #4 was analyzed using cDNA primers MLH1_promoter_cDNS_F and MLH1_promoter_cDNS_R (Table S1E). *MLH1* c.306+1222 A > G variant was confirmed from gDNA using primers MLH1_int03_F and MLH1_int03_R (Table S1E). cDNA-based functional characterization of the *MLH1* c.306+1222 A > G variant in patient #4 was performed by primer pairs MLH1_C1-F and MLH1_C2-R, MLH1_c.-93G_allele_spec_F and MLH1_C1-R, MLH1_promoter_cDNS_F and MLH1_ex04-ex03_R, respectively (Tables S1B and S1E).

## Results

### Establishment of a multimodal molecular genetic diagnostic workflow in LS

**Fig. 1:**
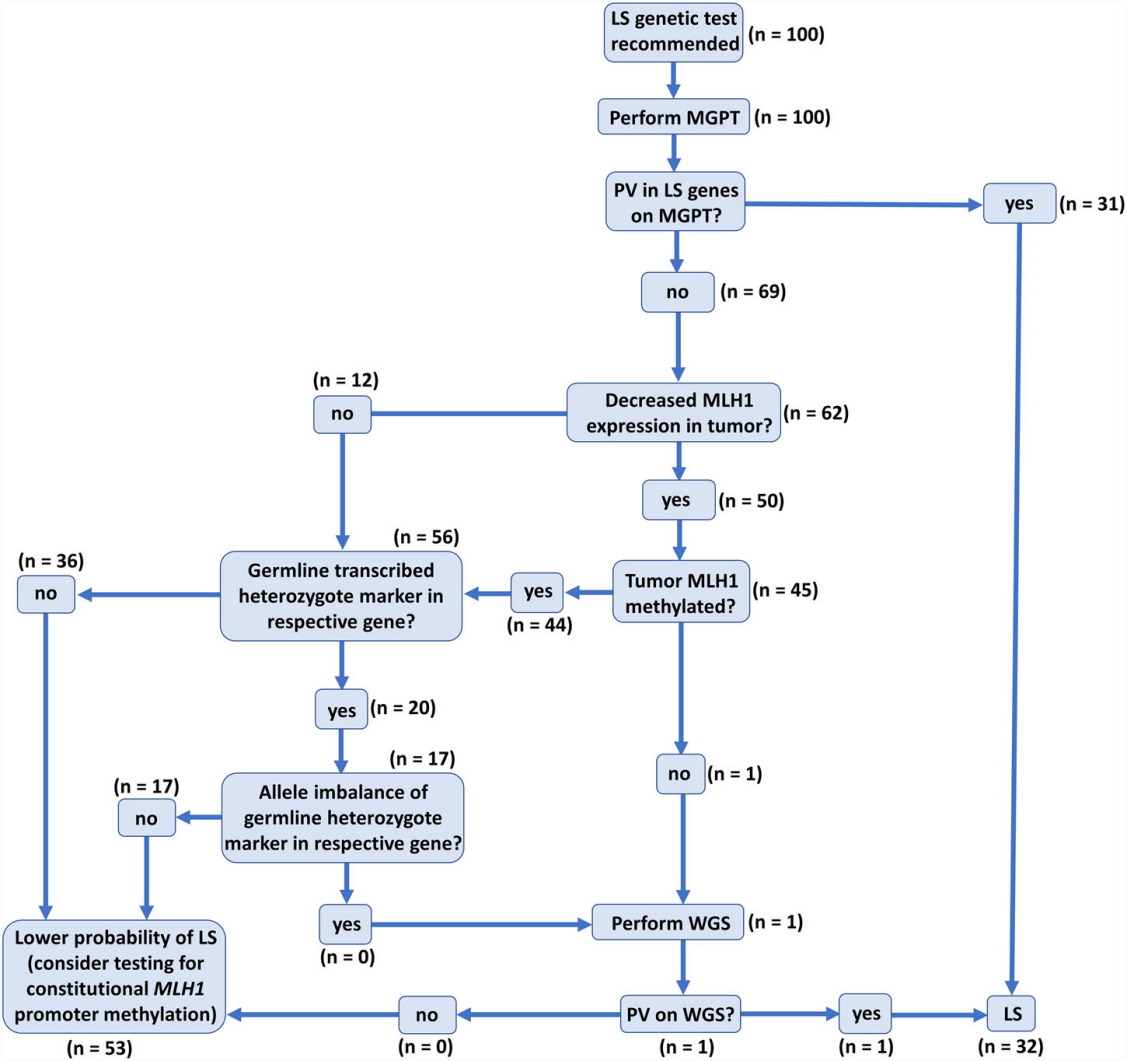
Molecular genetic diagnostic workflow for the diagnosis of LS. Numbers in brackets reflect the respective number of individuals in each node of the workflow. LS Lynch syndrome, MGPT multigene panel test, WGS whole genome sequencing.

The current NCCN guideline for LS testing recommends performing MGPT if testing criteria are met^8^. In cases where MGPT failed to detect a PV, a systematic screening is needed to prioritize samples for WGS. Availability of information regarding familial cancer background is highly variable and is irrelevant in the case of de novo PVs, therefore we opted for a prospective analysis of tumor tissue- and RNA-based methods (Fig. 1). If the reduced expression of MLH1 was confirmed in the pathological report, but the *MLH1* promoter was confirmed to be unmethylated in the tumor tissue our workflow recommended performing WGS. Otherwise, if an allelic imbalance of a germline heterozygote marker is confirmed in a gene with corresponding decreased protein expression in the tumor our workflow also advises performing WGS. This algorithm provides a fast, easy and cost-effective prioritization of samples for WGS.

### MGPT identifies complex PVs in LS genes

Thirty-one out of 100 investigated patients carried germline pathogenic or likely pathogenic variants in *MLH1* (*n* = 8), *MSH2* (*n* = 13), *MSH6* (*n* = 5), *PMS2* (*n* = 2) or *EPCAM* (*n* = 3) associated with LS (Fig. 1 and Table 1). Four out of these variants are novel SNVs (in patients #7 and #72) or SVs (in patients #10 and #97) with no previous records in the InSight or ClinVar databases.

**Table 1:** Clinical and molecular genetic characteristics of patients

patient ID	sex	age (years)	personal cancer history	familial (LS-associated) cancer history	dMMR/MSI phenotype	MGPT P/LP	InSight/LOVD		ClinVar	
							individual classifications	InSight classification	individual classifications	ClinVar classification
1	F	58	CRC58	—	dMMR (PMS2)					
2	F	50	CRC45	Paternal uncle CRC63	dMMR (MLH1, PMS2)					
3	M	33	CRC32	Father CRC29, mother CRC54, paternal grandmother CRC38, paternal grandfather PanC	dMMR (MLH1, PMS2)	*MLH1* c.1852_1854del p.(Lys618del)	P: 58, LP: 1, VUS: 81, NA: 18	Class 5, P	P: 24, LP: 1	P (reviewed by expert panel)
4*	F	62	CRC62	Mother CRC79, brother1CRC36, brother2BrainT42	dMMR (MLH1, PMS2)					
5	F	24	CRC24	—	dMMR (MSH2, MSH6)					
6	M	45	CRC45	—	dMMR (MLH1, MSH2)					
7	M	67	GC63, CRC67, PC67	Daughter EC37	dMMR (MSH2, MSH6)	***MSH2*** **c.592del p.(Glu198AsnfsTer16)**	**n.a**.	**n.a**.	**n.a**.	**n.a**.
8	F	68	CRC68	Paternal grandmother CRC65	dMMR (MLH1, PMS2)					
9	F	72	CRC72	—	dMMR (MLH1, PMS2)					
10	F	24	CRC22	Mother CRC51, maternal grandfather CRC	dMMR (MLH1, PMS2)	***MLH1*** c.[2078_2172del;2080_*+493dup]	**n.a**.	**n.a**.	**n.a**.	**n.a**.
11	M	31	CRC31	Cousin CRC33	MSI-H	*MLH1* c.298 C > T p.(Arg100Ter)	P: 16, VUS: 20, NA: 2	Class 5, P	P: 16	P (reviewed by expert panel)
12	F	64	CRC64	—	dMMR (MLH1, PMS2)					
13	M	59	CRC59	—	dMMR (MLH1, PMS2, MSH2)					
14	M	66	CRC66	Mother CRC72	dMMR (MLH1, PMS2)					
15	F	70	CRC70	—	dMMR (MLH1, PMS2)					
16	F	61	CRC61	—	dMMR (MLH1, PMS2)					
17	M	42	CRC37	—	dMMR (MLH1)					
18	M	77	PC65, CRC77	—	dMMR (MLH1, PMS2)					
19	F	70	CRC70	Paternal aunt GC89, paternal grnadfather GC82	dMMR (MLH1, PMS2)					
20	M	70	CRC67, PanC70, CRC70	—	n.a.					
21	F	50	CRC50	Cousin CRC55	dMMR (MSH6)	*MSH6* c.3261del p.(Phe1088SerfsTer2)	P: 37, NA: 3	Class 5, P	P: 24, NA: 1	P (reviewed by expert panel)
22	F	40	CRC35	Mother CRC39, EC54, father BrainT65	dMMR (MLH1, PMS2)	*MLH1* c.380 G > A p.(Arg127Lys)	P: 3, LP: 1, VUS: 2	Class 5, P	P: 2, LP: 1	P (reviewed by expert panel)
23	F	43	CRC43	maternal grandfather GC49	dMMR (MSH2, MSH6)	*EPCAM* del(ex8-9)	n.a.	n.a.	n.a.	n.a.
24	M	39	CRC39	-	dMMR (MLH1, PMS2)					
25	F	62	CRC62	—	dMMR (MSH2, MSH6)					
26	M	44	TC38, CRC43	Paternal aunt CRC73, paternal cousin CRC56	n.a.					
27	M	46	CRC43	—	dMMR (MSH2, MSH6)					
28	F	60	EC52, CRC52	Sister CRC49, mother OC56, paternal grandmother CRC76, paternal cousin CRC50	dMMR (MLH1, PMS2)	*MLH1* c.1039-2 A > T p.(?)	P: 2, LP: 1, VUS: 1, NA: 2	Class 4, LP	LP: 2	LP (reviewed by expert panel)
29	F	72	CRC70	—	dMMR (MLH1, PMS2)					
30	F	67	EC53, CRC67	Mother CRC46, maternal aunt CRC41, maternal cousin OC30, maternal grandmother CRC38, GC58	dMMR (MLH1, PMS2)	*MLH1* c.350 C > T p.(Thr117Met)	P: 51, LP: 2, VUS: 62, B: 2, NA: 22	Class 5, P	P: 24, LP: 1	P (reviewed by expert panel)
31	M	64	CRC35, CRC50, CRC63	Mother CRC27, GC, EC, maternal uncle CRC39, maternal grandmother CRC, GC, maternal grandfather PC, son PanC32	n.a.	*MLH1* c.1489dup p.(Arg497ProfsTer6)	P: 30, LP: 1, VUS: 18, NA: 1	Class 5, P	P: 19, NA: 1	P (reviewed by expert panel)
32	F	46	CRC46	Paternal cousin BrainT	dMMR (MSH6)					
33	M	52	CRC51	Paternal grandmother GC, paternal grandfather CRC	dMMR (MSH2, MSH6)	*MSH6* c.3226 C > T p.(Arg1076Cys)	P: 2, LP: 11, VUS: 1, NA: 2	Class 4, LP	P: 3, LP: 20	LP (reviewed by expert panel)
34	M	70	PC58, CRC70	Maternal uncle PC, maternal grandfather PC	MSI-H					
35	M	41	CRC40	Mother CRC38, CR63, maternal aunt1 CRC58, maternal aunt2 CRC64, CRC68, maternal grandmother CRC66	dMMR (MSH2, MSH6)	*EPCAM* del(ex8-9)	n.a.	n.a.	n.a.	n.a.
36	F	67	CRC66	—	dMMR (MLH1, PMS2)					
37	F	34	CRC23	Father CRC36, paternal grandmother EC, CRC32	dMMR (MSH2)	*MSH2* c.1216 C > T p.(Arg406Ter)	P: 55	Class 5, P	P: 22	P (reviewed by expert panel)
38	M	34	CRC33	Mother OC47, maternal grandmother CRC	dMMR (MSH2)	*MSH2* c.[2042 A > C;2045 C > T] p.[(Gln681Pro;Thr682Ile)]	n.a.	n.a.	LP: 1	LP (criteria provided, single submitter)
39	F	33	CRC3	Father GC55	dMMR (MSH2, MSH6)					
40	M	56	CRC54	—	dMMR (MLH1, PMS2)					
41	F	68	GC45, KC62, CRC67	—	dMMR (MSH2, MSH6)	*MSH2* c.226 C > T p.(Gln76Ter)	P: 26	Class 5, P	P: 7	P (reviewed by expert panel)
42	M	41	CRC39, CRC40	Sister CRC43, EC44, father UC47, paternal grandfather GC50	dMMR (MSH2)	*MSH2* c.2090 G > T p.(Cys697Phe)	P: 32, NA: 10	Class 5, P	P: 3	P (reviewed by expert panel)
43	F	56	CRC56	Sister CRC36, nephew CRC37, father CRC49	dMMR (MSH2, MSH6)	*MSH2* del(ex3-7)	n.a.	n.a.	n.a.	n.a.
44	M	30	CRC30	Father CRC62, paternal grandmother EC46, paternal grandfather CRC72	dMMR (PMS2)	*PMS2* c.1281del p.(His428ThrfsTer20)	n.a.	n.a.	P: 2, LP: 1	P/LP (criteria provided, multiple submitters, no conflicts)
45	M	68	CRC62	Son CRC30, mother EC46, father CRC72	n.a.	*MSH2* c.1906G>C p.(Ala636Pro)	P: 59, NA: 4	Class 5, P	P: 17, NA: 1	P (reviewed by expert panel)
46	M	49	CRC48	—	dMMR (MLH1)					
47	M	33	CRC33	—	dMMR (MSH2, MSH6)	*MSH6* c.3513_3514del p.(Asp1171fs)	P: 7	Class 5, P	P: 10	P (reviewed by expert panel)
48	M	30	CRC30	Mother EC48, maternal grandmother CRC47, CRC53	n.a.	*MSH2* del(ex9-15)	n.a.	n.a.	n.a.	n.a.
49	M	67	CRC67	—	dMMR (MLH1)					
50	M	69	CRC69	Son1 CRC35, son2 CRC44, mother CRC58, maternal uncle1 CRC, maternal uncle2 CRC	dMMR (MSH2)	*EPCAM* del(ex8-9)	n.a.	n.a.	n.a.	n.a.
51	M	56	CRC56	Father CRC65, paternal uncle1 BrainT, paternal uncle2 PC70	dMMR (MLH1, PMS2)					
52	F	72	CRC72	—	dMMR (MSH2, MSH6)					
53	F	78	CRC77	n.a.	MSI-H					
54	F	63	CRC62	—	dMMR (MLH1, PMS2)					
55	F	55	CRC41, EC50, CRC55	Mother CRC35, EC	MSI-H	*MSH2* c.1165 C > T p.(Arg389Ter)	P: 53, NA: 1	Class 5, P	P: 20, NA: 1	P (reviewed by expert panel)
56	F	49	CRC46	—	dMMR (MSH2)					
57	M	61	CRC61	Maternal uncle GC80	dMMR (MSH2, MSH6)					
58	M	42	CRC42	—	MSI-H					
59	F	67	CRC67	Mother CRC68	MSI-H					
60	F	68	CRC68	Maternal aunt CRC57, maternal uncle CRC60, maternal grandmother GC65	dMMR (MLH1, PMS2)					
61	F	64	CRC62	Mother KC47, maternal uncle1 CRC, maternal uncle2 CRC, maternal grandmother CRC80	dMMR (MLH1, PMS2)					
62	F	62	CRC61	Maternal aunt KC	dMMR (MLH1, PMS2)					
63	F	74	EC73	Father GC	dMMR (MSH6)	*MSH6* c.114del p.(Ala40ProfsTer41)	n.a.	n.a.	P: 3	P (criteria provided, multiple submitters, no conflicts)
64	F	36	EC36	Father CRC43, paternal unlce GC37, patrnal grandfather GC53, paternal grnadmother OC60	dMMR (MSH2, MSH6)	*MSH2* c.2068 C > T p.(Gln690Ter)	n.a.	n.a.	P: 1	P (criteria provided, single submitter)
65	F	68	UC47, lymphoma48, EC68	—	dMMR (MLH1, PMS2)					
66	F	66	CRC56, EC67	Mother PanC63	dMMR (MLH1, PMS2)					
67	F	38	EC37	Sister EC37, mother EC42, CRC47, maternal grandmother GC, paternal grandfather GC	dMMR (PMS2)	*MLH1* c.1210_1211del p.(Leu404ValfsTer12)	P: 2, VUS: 2	Class 5, P	P: 6	P (reviewed by expert panel)
68	F	77	EC76	—	dMMR (MLH1, PMS2)					
69	F	64	EC64	Mother CRC52, paternal grandfather GC	dMMR (MLH1, PMS2)					
70	F	70	EC70	—	dMMR (MLH1, PMS2)					
71	F	78	EC78	—	dMMR (MLH1, PMS2)					
72	F	40	EC39	—	dMMR (MSH2, MSH6)	***MSH2*** **c.2361_2364dup p.(Ala789TyrfsTer11)**	n.a.	n.a.	n.a.	n.a.
73	F	65	EC65	Mother BrainT73, maternal cousin BrainT54	dMMR (MLH1, PMS2)					
74	F	57	EC56	—	dMMR (MLH1, PMS2)					
75	F	42	EC42	—	dMMR (MSH6)					
76	F	50	EC50	Maternal aunt OC71	dMMR (MLH1, PMS2)					
77	F	64	EC64	—	dMMR (MLH1, PMS2)					
78	F	70	EC70	—	dMMR (MLH1, PMS2)					
79	F	52	EC52	Mother PanC79	dMMR (MLH1, PMS2)					
80	F	54	EC54	Maternal uncle BrainT74	dMMR (MSH6)					
81	F	61	EC61	Mother CRC65	dMMR (MLH1, PMS2)					
82	F	79	EC75	Sister EC75, maternal aunt EC, maternal cousin1 CRC, maternal cousin2 UC, maternal cousin3 UC	dMMR (MLH1, PMS2)					
83	F	72	EC72	—	dMMR (MLH1, PMS2)					
84	F	72	EC72	Father PC74, maternal aunt EC73	dMMR (MLH1, PMS2)					
85	F	72	EC72	Paternal aunt CRC	dMMR (MLH1, PMS2)					
86	F	61	EC61	Paternal aunt BrainT59	dMMR (MLH1, PMS2)					
87	F	64	EC63	Maternal aunt CRC86, maternal aunt CRC87, maternal cousin PanC67	dMMR (MLH1, PMS2)					
88	F	62	EC62	Father CRC88	dMMR (MSH2, MSH6)					
89	F	61	EC61	—	dMMR (MLH1, PMS2)					
90	F	66	EC66	Paternal uncle CRC62	dMMR (MLH1, PMS2)					
91	F	57	EC56	—	MSI-H					
92	F	37	EC36	Father CRC60, paternal grandfather PanC55, paternal grandmother EC53, maternal grandfather BrainT65	MSI-H	*MSH6* c.3959_3962del p.(Ala1320GlufsTer6)	P: 10, NA: 1	Class 5, P	P: 18, NA: 2	P (reviewed by expert panel)
93	F	57	CRC46, EC56	Mother CRC68, UC69, PanC71, maternal aunt1 CRC60, maternal aunt2 CRC, maternal grandmother CRC77, paternal uncle CRC72, paternal grandmother CRC87	dMMR (MSH2, MSH6)	*MSH2* c.2494 G > T p.(Glu832Ter)	P: 1	n.a.	P: 1, LP: 1	P (criteria provided, single submitter)
94	F	61	EC59	Mother KC67, paternal grandfather GC	dMMR (MLH1, PMS2)					
95	F	69	EC69	—	dMMR (MLH1, PMS2)					
96	F	66	EC66	Sister OC77, brother PanC, maternal uncle PanC49	dMMR (MLH1, PMS2)					
97	F	58	EC58	—	dMMR (MSH2, MSH6)	***MSH2*** **c.2506_2507ins[G;2507_2620]**	n.a.	n.a.	n.a.	n.a.
98	F	66	CRC62, EC66	Mother EC36, maternal cousin1 CRC, maternal cousin2 EC, maternal cousin3 KC, paternal cousin CRC	dMMR (MSH6, PMS2)	*PMS2* del(ex1-15)	n.a.	n.a.	n.a.	n.a.
99	F	70	EC70	Brother GC, maternal grandmother EC62, maternal cousin PC58	dMMR (MLH1, PMS2)					
100	F	54	EC54	Mother PanC66	dMMR (MLH1, PMS2)					

Variants correspond to the MANE Select transcript of each gene (*MLH1*: NM_000249.4; *MSH2*: NM_000251.3; *MSH6*: NM_000179.3; *PMS2*: NM_000535.7; *EPCAM*: NM_002354.3). *M* male, *F* female, *IHC* immunohistochemistry, *CRC* colorectal cancer, *EC* endometrial cancer, *UC* urothelial cancer, *PanC* pancreatic cancer, *PC* prostate cancer, *GC* gastric cancer, *TC* testicular cancer, *BrainT* brain tumor, *OC* ovarian cancer, *KC* kidney cancer, *dMMR* mismatch repair deficiency, *MGPT* multigene panel test, *MSI-H* high levels of microsatellite instability, *P* pathogenic, *LP* likely pathogenic, *VUS* a variant of unknown significance, *B* benign, *NA* not applicable, *P/LP* pathogenic or likely pathogenic variants. Previously unreported SNVs and SVs are highlighted in bold letters. Asterisk mark patient who was diagnosed with an *MLH1* deep intronic pathogenic variant during follow-up whole genome sequencing. Note: Patient #44 is the son of patient #45. Note: Two transcripts are derived from the SV found in patient #10.

**Fig. 2:**
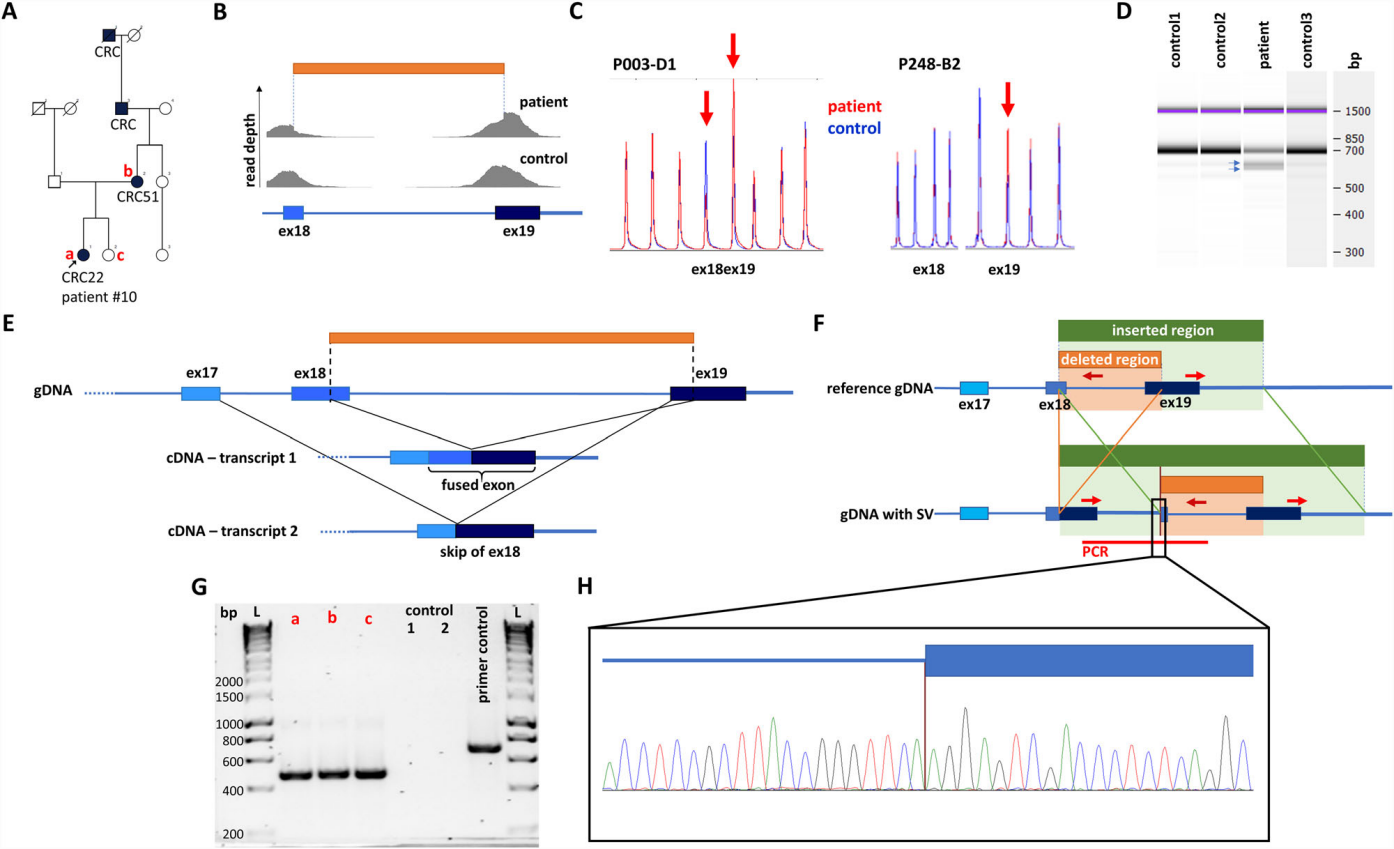
Characterization of a novel *MLH1* SV in patient #10. Pedigree of the proband (**A**). Visual representation of detected reads of exons 18 and 19 in the BAM file (**B**). MLPA analyses with default (P003-D1) and confirmation (P248-B2) probe sets, extended data can be found in Figure S1 (**C**). Electrophoretic separation of *MLH1* cDNA amplicons on an Agilent Bioanalyzer DNA1000 chip (**D**). Arrows point to two additional cDNA transcripts. Note: Uncropped image is demonstrated in Figure S2. Visual representation of the architecture of the two additional cDNA transcripts detected with Sanger sequencing (**E**). Genomic architecture of the detected SV compared to the reference gDNA (**F**). Breakpoint-specific PCR primers are visualized as red arrows binding at sequence complementary regions. Primers oriented outwards in the reference genome get face each other only at the duplication breakpoint. Electrophoretic separation of breakpoint-specific PCR products on 1.5% agarose gel (**G**) performed on germline DNA sample of the proband (a), her mother (b), her sister (c) and two independent controls. Note: a, b and c individuals are highlighted also in Panel **A**. Note: Uncropped image is demonstrated in Figure S3. Chromatogram visualizing the breakpoint (vertical brown line) included in the breakpoint-specific PCR (**H**). PCR polymerase chain reaction, L ladder.

In patient #10, we identified an SV affecting *MLH1*. The proband developed colorectal cancer (CRC) at the age of 22 years, with familial predisposition and molecular pathological analysis (decreased MLH1 and PMS2 protein expressions in both the proband and her mother’s CRC) both indicative of LS (Table 1 and Fig. 2A). Primary bioinformatic analysis performed on the DRAGEN SV pipeline signaled a potential complex alteration (Table S2) in *MLH1*. Both SV call and visual inspection of the BAM file reads suggested a 1563 base pair (bp) deletion (Fig. 2B). MLPA analysis (probe set: P003-D1 MLH1/MSH2) revealed a deletion of exon 18 and a duplication of exon 19 (Fig. 2C, Figure S1). Confirmatory MLPA analysis with a different MLPA probe set (probe set P248-B2 MLH1-MSH2 Confirmation) only corroborated the duplication of exon 19 (Fig. 2C, Figure S1). To further characterize the transcriptional consequence of this variant, RNA-based analyses have been performed. Two additional, shorter transcripts were detected on the cDNA level (Fig. 2D). Sanger sequencing of these amplicons revealed one of them to include the fusion exon between exons 18 and 19, which was imputed from the deletion observed in the NGS data, however, the other altered transcript lacked the whole exon 18, while exon 19 was present in its entirety (Fig. 2E). Based on these observations we hypothesized that the complex alteration consisted of a tandem duplication of a larger region stretching from the 3’ part of exon 18 and the neighboring regions containing the whole exon 19 and a partial deletion of the 5’ copy of this duplication including the 3’ part of exon 18 and the 5’ part of exon 19 (Fig. 2F). To confirm this hypothesis, a breakpoint-specific PCR has been designed with the forward primer binding to exon 19 and the reverse primer binding the interconnecting intron of exons 18 and 19 (Fig. 2F-G). Successful and specific amplification of the amplicon including the breakpoint in the proband and her relatives carrying this SV not only confirmed this hypothesis but also allowed the precise nomenclature of the variant as *MLH1* (NM_000249.4) c.[2078_2172del; 2080_*+493dup] (Fig. 2H).

**Fig. 3:**
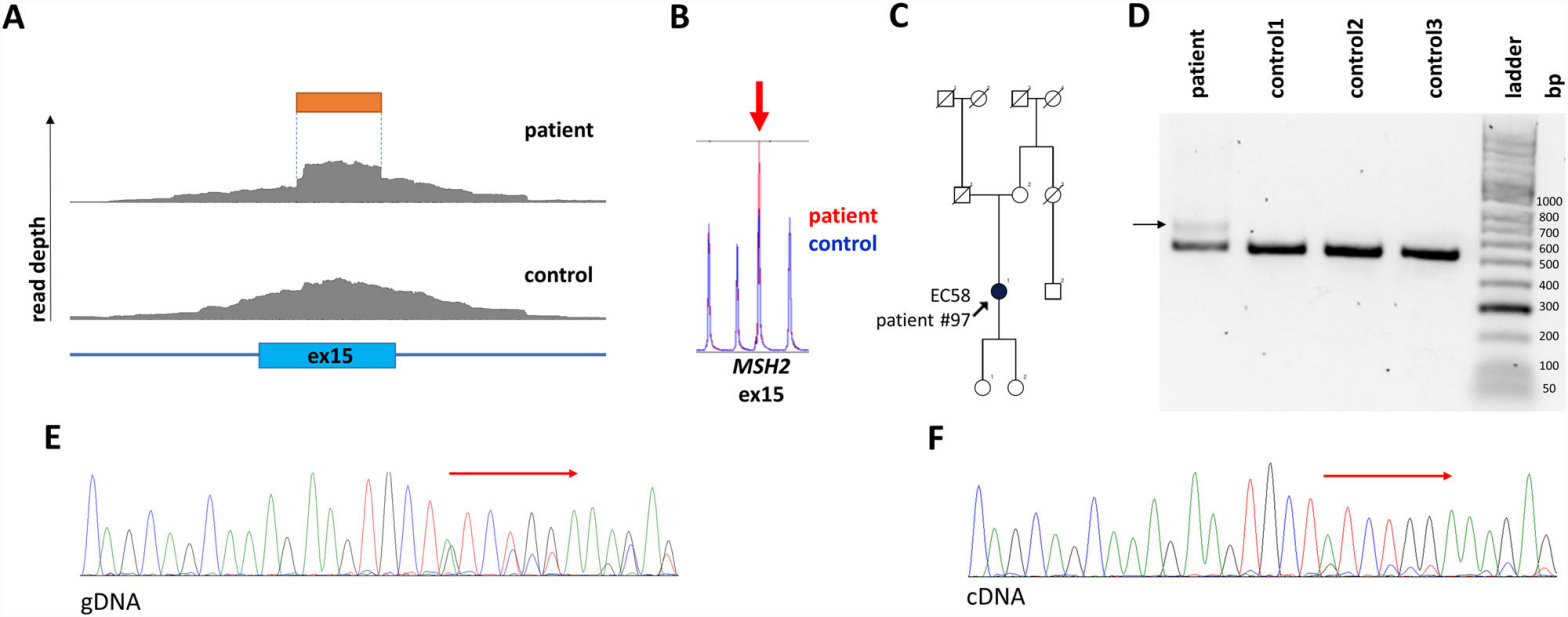
Analysis of *MSH2* c.2620_2621ins[G;2507_2620] in patient #97. Visual representation of the partial duplication of exon 15, contributing to the SV in the BAM file observed in patient #97 (**A**). MLPA analysis (probe set #P003-D1) extended data can be found in Figure S4 (**B**). Pedigree of patient #98 (**C**). Electrophoretic separation of *MSH2* cDNA amplicons on a 1.5% agarose gel (**D**). The arrow point to an additional cDNA transcript. Note: Uncropped image is demonstrated in Figure S5. Chromatogram of gDNA-based Sanger sequencing visualizing the 5’ start of the detected SV (**E**). Chromatogram of cDNA-based Sanger sequencing visualizing the 5’ start of the detected SV (**F**). bp base pair, EC endometrial cancer.

In patient #97 we identified an SV in *MSH2* consisting of an insertion of a guanine and a duplication of the 5’ 114 bps from the insertion (NM_000251.3 c.2620_2621ins[G;2507_2620], Fig. 3A). MLPA also pointed out the SV (as a duplication of exon 15), since the probe hybridization sequence fell into the duplicated stretch (Fig. 3B and Figure S4). This variant was found in a woman with a prior diagnosis of endometrial cancer at the age of 58 years, while no familial cancer history was explorable (Fig. 3C). Immunohistochemical analysis of the tumor revealed a tumor-specific decrease in the expression of MSH2 and MSH6, corresponding to the germline PV. RNA-based analyses confirmed the same sequence context as experienced in the germline: the insertion was faithfully transcribed, without any interference with splicing (r.2620_2621ins[G;2507_2620]). However, since the deduced translation product contained a premature termination codon (p.Tyr874Cysfs*3), the altered transcript occurred at a lower abundance probably as a result of nonsense-mediated decay (NMD, Fig. 3D-F).

**Fig. 4:**
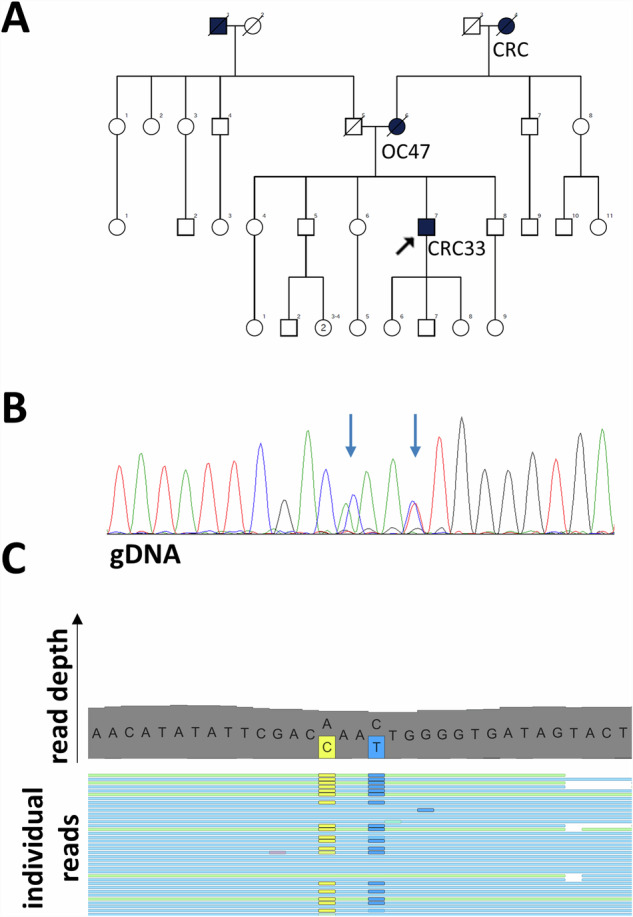
Analysis of the *MSH2* c.[2042 A > C;2045 C > T] variant in patient #38. Pedigree of patient #38 (**A**). Chromatogram of gDNA-based Sanger sequencing visualizing the complex PV *MSH2* c.[2042 A > C;2045 C > T] (**B**). The arrow points to the heterozygous loci of c.2042 A > C and c.2045 C > T. Representative image of reads revealing the co-occurrence of c.2042 A > C and c.2045 C > T on the same allele in the BAM file (**C**). Yellow boxes represent the alternative C at the 2042 position, while blue boxes represent the alternative T at the 2045 position.

In addition to the detected SVs, we have found a third complex PV in patient #38 as a result of two rare SNVs within 4 base pairs in *MSH2*. The proband was diagnosed with MSH2- and MSH6-deficient colorectal cancer at the age of 33 years with more LS-related cancer occurrences in the pedigree (Fig. 4A). MGPT followed by Sanger sequencing validated two SNVs within 4 base pairs in the *MSH2* gene (Fig. 4B) which were confirmed to be on the same allele (Fig. 4C). As HGVS nomenclature (version 21.0.2, https://hgvs-nomenclature.org/stable/) requires the independent description of such variants with the signal that they are in *cis* position, the adequate designation is *MSH2* c.[2042 A > C;2045 C > T], p.[(Gln681Pro; Thr682Ile)]. We have found no previous record with this naming in the ClinVar or Insight databases, however, the alternative naming of c.2042_2045delinsCAAT revealed an earlier record with likely pathogenic classification. Additionally, functional testing of the c.2042 A > C variant altering codon 681 revealed a deleterious effect on protein function^15^.

### Somatic *MLH1* promoter methylation and RNA-based analyses prioritize sample for WGS

**Fig. 5:**
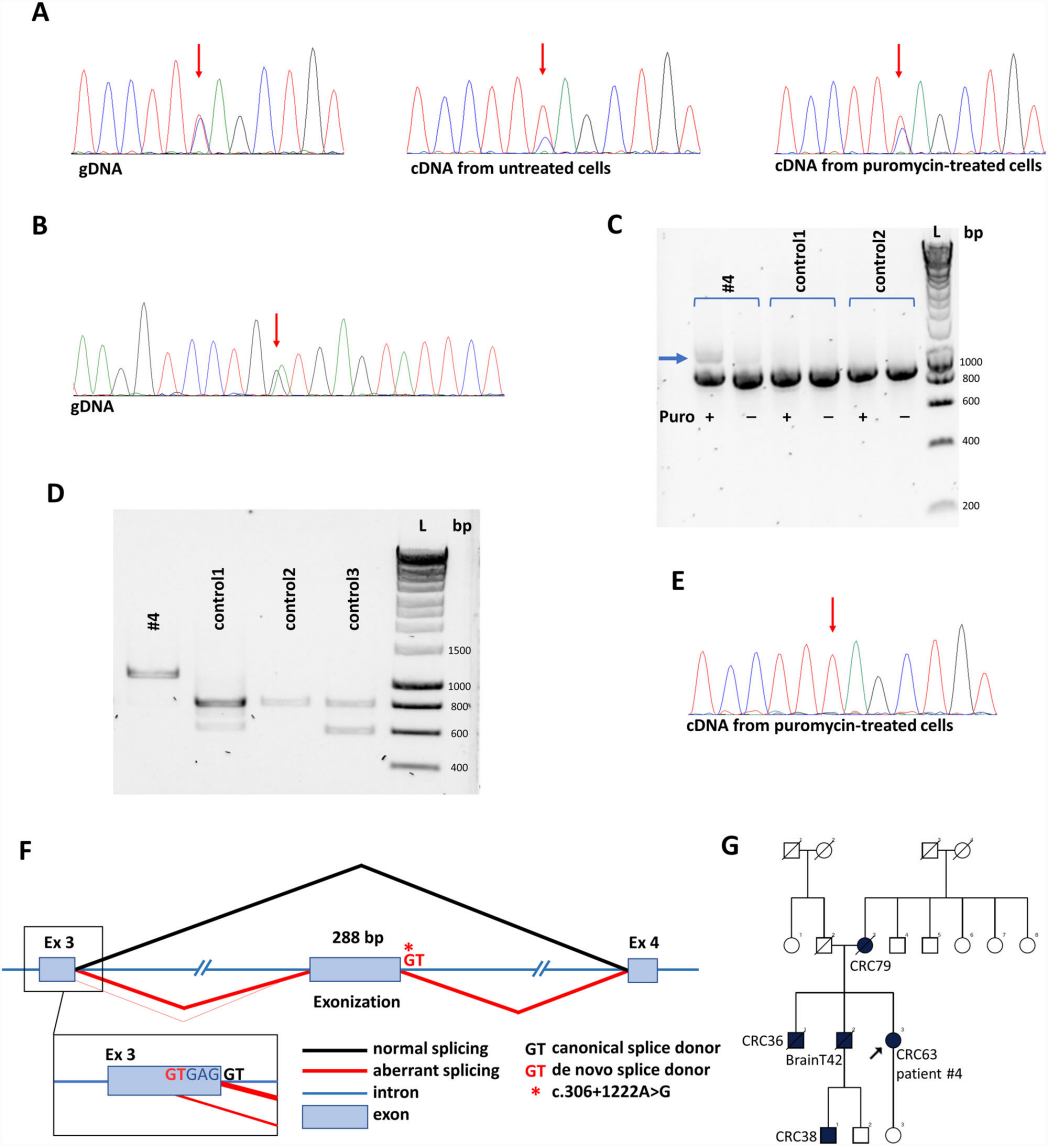
WGS-based detection and characterization of a novel deep-intronic PV in *MLH1* in patient #4. Allele imbalance detected at the *MLH1* c.-93G > A heterozygote marker in patient #4 (**A**). Note: results from the reverse sequencing are presented. Puromycin treatment almost restored the allelic ratios measured at the gDNA-level. Red arrows show the position of *MLH1* c.-93G > A. Chromatogram of gDNA-based Sanger sequencing of the *MLH1* c.306+1222 A > G variant (**B**). The arrow points to the heterozygous locus of c.306+1222 A > G. Electrophoretic separation from *MLH1* cDNA amplicons incorporating exons 1-9 from patient #4 and controls (**C**). Puromycin (Puro) treatment inhibits NMD. Note: Uncropped image is demonstrated in Figure S6. Electrophoretic separation of *MLH1* cDNA amplicons restricted from alleles containing the reference ’G’ base at *MLH1* c.-93 position (**D**). This allowed the amplification to occur from the allele which carried the detected deep intronic variant, as the alternative ’G’ base at the c.306+1222 position was in phase with the reference ’G’ base at the c.-93 position in patient #4 (as highlighted in **A**). Note: Uncropped image is demonstrated in Figure S7. Chromatogram of cDNA-based Sanger sequencing restricted from wild type cDNA transcript highlighting the -93G > A position from patient #4 (**E**). Restriction for the wild-type cDNA transcript was allowed by the reference primer which was specific to the exon 3-4 boundary. Note: results from the reverse sequencing are presented and might be compared to those presented in (**A**). The red arrow shows the position of *MLH1* c.-93G > A. Genomic architecture of altered splicing resulting from the deep intronic variant *MLH1* c.306+1222 A > G (**F**). Pedigree of patient #4 (**G**). bp base pair, BrainT brain tumor, CRC colorectal cancer, L ladder, NMD nonsense-mediated decay, Puro puromycin.

As per our recommended workflow (Fig. 1), PV-negative cases were further studied to select samples for WGS. *MLH1* promoter methylation is a frequent epigenetic mechanism responsible for decreased MLH1 expression in tumors. In our cohort, 45 out of 50 tumor tissues with decreased expression of MLH1 were available for testing for *MLH1* promoter methylation, among which all but one sample exhibited the methylation in all 16 analyzed CpG positions. The tumor sample of patient #4 was unmethylated at the *MLH1* promoter and thus was selected for WGS. Allelic imbalance in the RNA level was used as another prioritization method to select samples for WGS, as both pathogenic promoter or deep intronic variants resulting in frameshift proteins and NMD can cause the relative predominance of the reference allele. No presentation of allelic imbalance was observed among the 17 cases, where germline transcribed heterozygote markers were present in the adequate mismatch repair gene (i.e. the one coding the protein with decreased expression in the tumor). However, the sample of patient #4 already selected for WGS exhibited a significant allelic imbalance regarding the promoter variant *MLH1* c.-93G > A, which was due to NMD, as inhibition of NMD diminished this effect (Fig. 5A).

### WGS detects a deep intronic PV in *MLH1*

WGS analysis of the gDNA of patient #4 detected 12 variants in *MLH1* and further filtering regarding putative splice alterations revealed a rare SNV c.306+1222 A > G located in intron 3 (Fig. 5B, Supplementary Data S1). This variant was predicted to activate a deep intronic cryptic splice donor site, resulting in the exonization of an intronic sequence. cDNA amplification of the *MLH1* amplicon incorporating exons 1–9 revealed an additional, larger PCR product in NMD-inhibited cells, which was absent in controls (Fig. 5C). We confirmed the pathogenicity of this deep intronic variant in two ways. Both ways harnessed marker variants, the phase of which relative to the studied intronic variant was determined by the former molecular analyses (i.e. allelic imbalance studies). First, we performed allele-specific RT-PCR restricted from the intronic variant-carrying allele and revealed the exclusive amplification of an altered mRNA incorporating a 288 bp-long intronic sequence upstream of c.306+1222 A > G (Fig. 5D). Second, we applied a so-called tagging variant analysis, where RT-PCR amplicon was generated with a reverse primer specific to the exon 3–4 boundary (and therefore selecting for the wild-type mRNA). The sequencing chromatogram of this amplicon did not contain the tagging variant (which is in phase with c.306+1222 A > G), so it revealed that reference mRNA is derived exclusively from the reference gDNA allele (Fig. 5E). The main aberrant transcript was r.306 + 934_306+1221ins, but to a much lesser extent r.[301_305del;306 + 934_306+1221ins] was also present (Fig. 5F). The inserted intronic sequence causes frameshift and consequential premature termination codon, so elicits NMD mechanism, which correlates very well with the strong allelic imbalance detected in this patient. The confirmation of its transcriptional consequence allowed the correct genetic diagnosis in a family with multiple LS-associated cancer manifestations (Fig. 5G), where the proband was diagnosed with MLH1-deficient colorectal cancer (Table 1, Fig. 5G). The inclusion of WGS following a prioritization workflow elevated the detection rate of PVs resulting in LS from 31% to 32% (Fig. 1A).

### Recurring complex PVs in LS

**Fig. 6:**
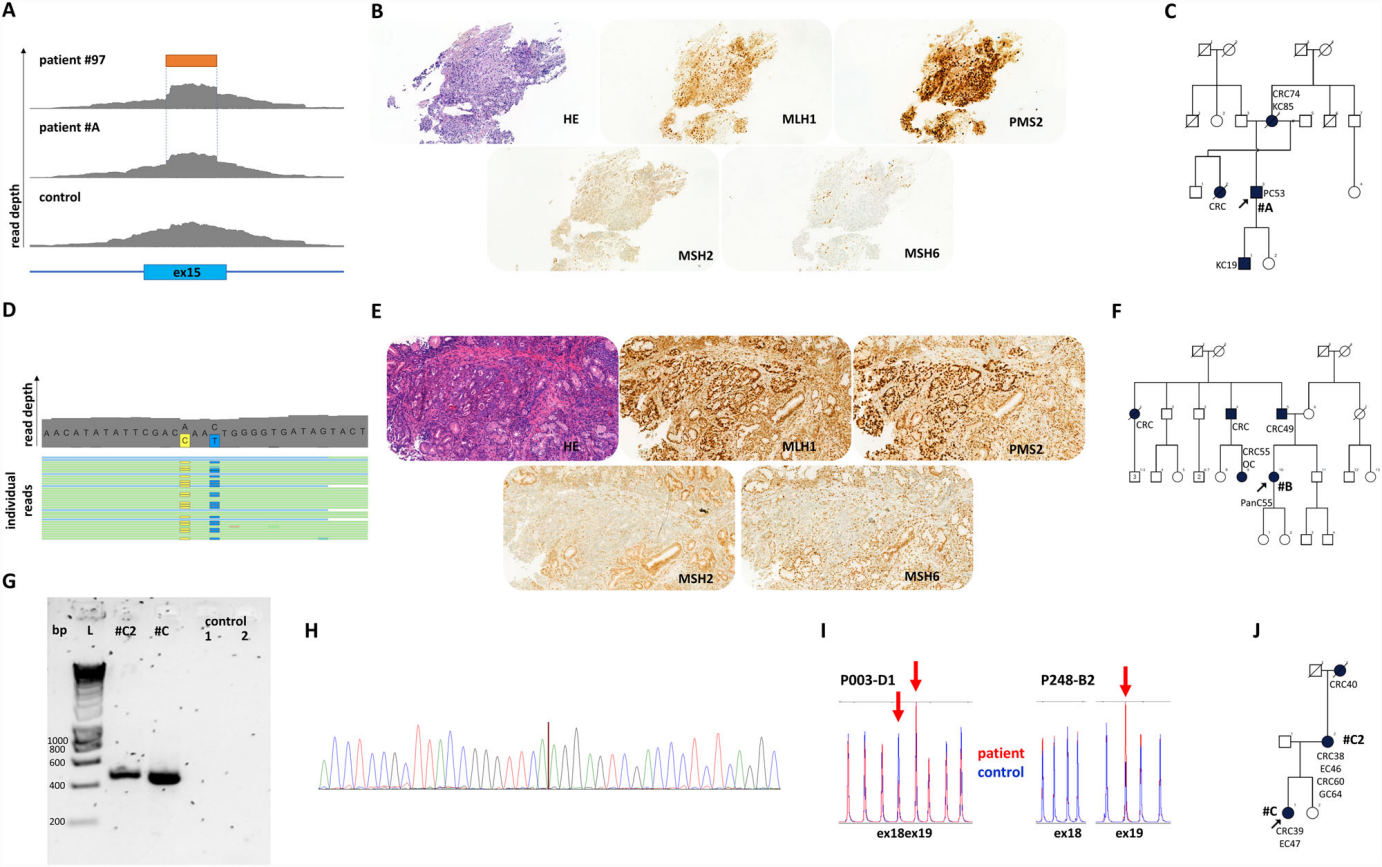
Recurrent complex PVs in additional families. Visual representation of the partial duplication of *MSH*2 exon 15, contributing to the SV in the BAM file observed in patient #A as well as patient #97 (**A**). Note: Read depths of patient #97 and control are also demonstrated in Fig. 4A and are presented here for optimal comparison. Immunohistochemical analysis of the mismatch repair proteins in the prostate cancer biopsy of patient #A (**B**). 20× magnification. Pedigree of patient #A (**C**). Representative image of reads revealing the co-occurrence of *MSH2* c.2042 A > C and c.2045 C > T on the same allele from the BAM file in patient #B (**D**). Yellow boxes represent the alternative C at the 2042 position, while blue boxes represent the alternative T at the 2045 position. Immunohistochemical analysis of the mismatch repair proteins in the pancreatic cancer specimen of patient #B (**E**). 20× magnification. Pedigree of patient #B (**F**). Electrophoretic separation of *MLH1* c.[2078_2172del; 2080_*+493dup]-specific PCR (further elaborated in Fig. 2F–H) products on 1.5% agarose gel (**G**) performed on germline DNA sample of patients #C and #C2 and two independent controls. Note: patients #C and #C2 are highlighted also on Panel J. Note: Uncropped image is demonstrated in Figure S8. Chromatogram visualizing the breakpoint (vertical brown line) included in the breakpoint-specific PCR (**H**). MLPA analyses with default (P003-D1) and confirmation (P248-B2) probe sets (**I**). Pedigree of patient #C (**J**). bp base pair, CRC colorectal cancer, EC endometrial cancer, GC gastric cancer, KC kidney cancer, L DNA ladder, OC ovarian cancer, PanC pancreatic cancer, PC prostate cancer.

To determine if the detected complex PVs were present in independent families within the analyzed time frame, we examined if there were any recurrent PVs in other patients who underwent genetic counseling and testing at our center. The *MSH2* SV found in patient #97 (c.2620_2621ins[G;2507_2620]) was detected in a patient (patient #A) with prostate cancer (Fig. 6A) and lack of MSH2 and MSH6 expression in the tumor tissue verified the pathogenic nature of this variant concerning this malignancy (Fig. 6B). Interestingly, this family exhibited an abundance of urinary tract cancers (Fig. 6C) which is in line with the recently reported higher risk of urinary tract cancers in the carriers of *MSH2* PVs^16^. Since this partial exon duplication results in framsehift and premature stop codon (PVS1 – very strong), confirmed by the functional mRNA-based assay presented in Fig. 3D-F (PS3 – strong) and we have identified this variant in two patients with a phenotype highly specific to *MSH2*-associated LS (PP4 – supporting), the ACMG classification of this variant is pathogenic.

The *MSH2* c.[2042 A > C;2045 C > T] was also identified in patient #B, where the two variants were also found on the same allele (Fig. 6D). Patient #B was diagnosed with pancreatic cancer at the age of 55 and immunohistochemical analyses on the tumor tissue revealed the pathogenic nature of this variant concerning this rare LS manifestation (Fig. 6E). The patient’s familial cancer history included 4 diagnosed CRCs and one ovarian cancer (Fig. 6F). As in vitro data strongly support the pathogenicity of *MSH2* c.2042 A > C^15^ fulfilling the criteria PS3 (strong), the variant is absent from the studied gnomAD database (PM2—moderate) and we have identified it in two patients with a phenotype highly specific to *MSH2*-associated LS (PP4 – supporting), the ACMG classification of this variant is likely pathogenic.

Our research group has previously reported an LS family with a germline *MLH1* deletion affecting the same region (*MLH1* c.2078_2172del) as observed in patient #10^17^. We have re-evaluated the gDNA samples of this previous family (HFC100 in the original publication, patient #C in the current study) and have confirmed that they harbor the same complex SV c.[2078_2172del; 2080_*+493dup] as patient #10 (Fig. 6G-J and Table S3). Additionally, following the completion of patient accrual to our pilot study, we have detected this complex *MLH1* SV in a third independent family (patient #D, Table S3). The proband was diagnosed with three primary LS-associated tumors (one endometrial cancer at the age of 55 years and two CRCs (at the ages of 54 and 59 years), immunohistochemical analysis was available from the latter, which confirmed the decreased expression of MLH1 and PMS2). As this SV affects multiple exons resulting in a frameshift mutation (PVS1—very strong), we have confirmed the functional consequence on the mRNA level (PS3 – strong) and have confirmed the occurrence of the variant in three independent families with a clinical diagnosis of LS, among which 3 CRCs from two families exhibited the highly specific phenotype (MLH1- and PMS2-deficient CRC), satisfying the PP4 criteria (supporting), the ACMG classification of this variant is pathogenic.

Although limited segregation analyses were available in the family of patient #10, they were unavailable to assess in the families of patients #38, #97, #A and #B. Additionally, it is important to note, that proper segregation analyses necessitate the investigation of multiple first- second- and third-degree family members to confirm cosegregation^18^. In cases, where these segregation analyses were not available, the pathogenicity of the detected variants was identified by fulfilling additional criteria of the ACMG guideline^9^ as specifically described above. Although cascade testing was offered to 137 first-degree family members of the 32 patients diagnosed with LS by MGPT, only 34 family members (24.8%) chose to undergo genetic testing. This correlates with larger studies finding the request for cascade testing to be between 10% and 30%^19^ and represent a limitation in assessing pathogenicity.

In summary, all of the complex PVs identified with MGPT were confirmed to be recurrent in our investigated population.

### WGS analysis of a retrospective cohort of PV-negative patients reveals the recurrent nature of *MLH1* c.306+1222 A > G

**Fig. 7:**
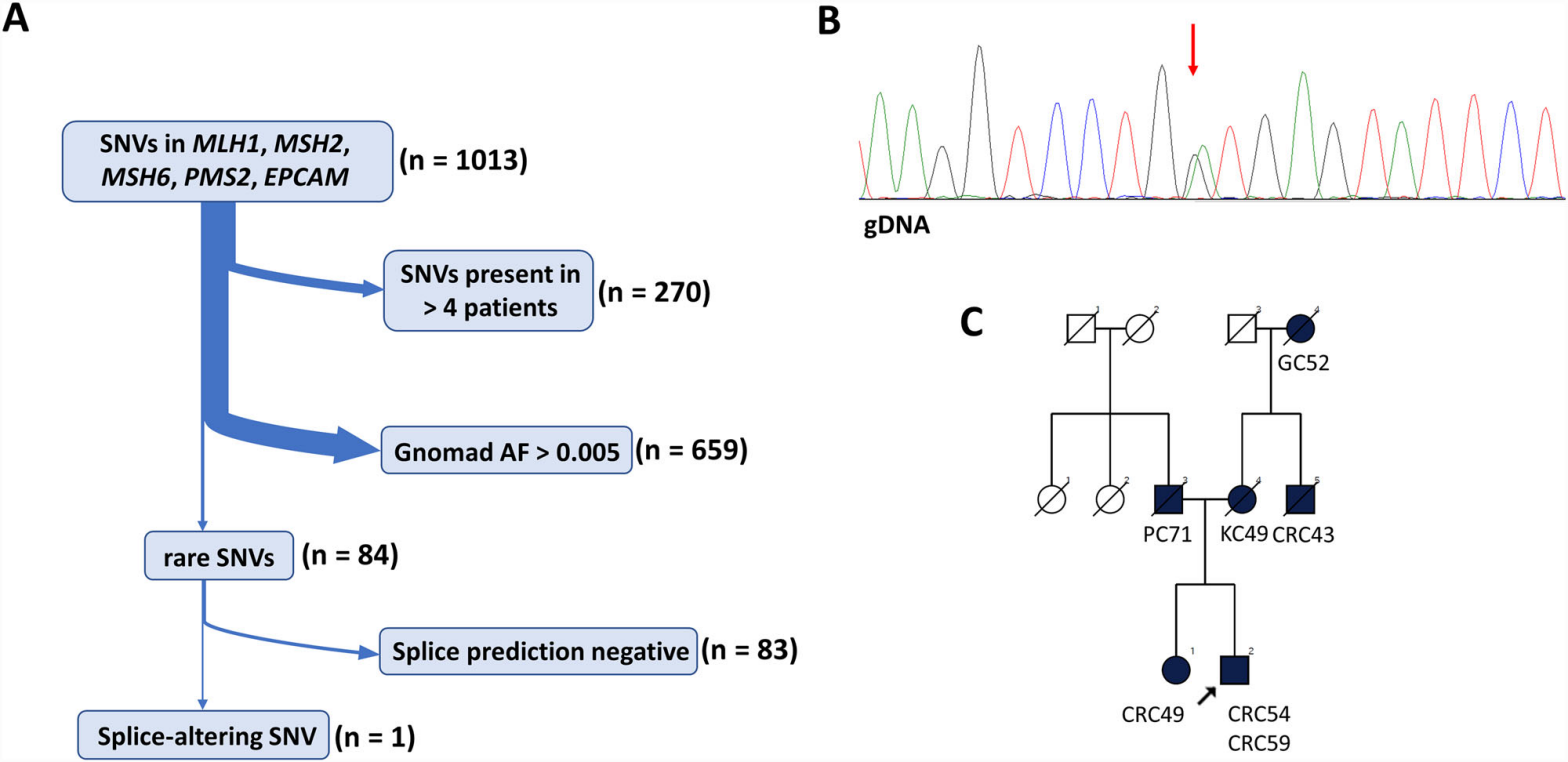
Retrospective analysis of 10 patients with WGS. Filtering strategy of SNV (**A**). Chromatogram of gDNA-based Sanger sequencing visualizing the *MLH1* c.306+1222 A > G variant in patient #W10 (**B**). Pedigree of patient #W10 (**C**). AF allele frequency, CRC colorectal cancer, GC gastric cancer, KC kidney cancer, PC prostate cancer, SNV single nucleotide variant.

Additionally, we performed WGS in a retrospective cohort from our center with a high likelihood of Lynch syndrome, where previous genetic analyses found no PVs (Table S4). We found no SVs or CNV in the genes of *MLH1*, *MSH2*, *MSH6*, *PMS2* and *EPCAM*, however, 1013 SNVs were called. Further filtering of the variants excluded frequent variants and finally computational splice-altering predictions were performed (Fig. 7A, Supplementary Data S2). This workflow returned the same *MLH1* deep intronic variant previously found in patient #4 (*MLH1* c.306+1222 A > G) in patient #W10 (Fig. 7B-C), who was first diagnosed with MLH1- and PMS2-deficient CRC at the age of 54 years. As we were able to confirm the damaging functional consequence of this variant in patient #4 (PS3 – strong), this variant is not present in gnomAD referenc database (PM2 – moderate) and both the affected patients’ phenotype are highly specific to *MLH1*-associated LS (PP4 – supporting), the ACMG classification of this variant is likely pathogenic.

As we have identified the *MLH1* c.306+1222 A > G variant in our retrospective cohort as well, we were able to confirm the recurrent nature of this variant in our population.

## Discussion

The diagnosis of LS has rapidly evolved in the past decades. In 2004, the revised Bethesda guidelines advised microsatellite instability (MSI) testing of only high-risk CRCs^7^, while today all CRCs are recommended to be tested for MSI^20^. The revised Bethesda guidelines advised germline testing only if mismatch repair deficiency has been confirmed^7^, while the latest NCCN guideline does not require prior testing for MSI in young CRC patients for germline testing^8^ and advocates for universal germline testing argue for offering genetic testing for all CRC patients^21–23^. On the technical side, the wide-ranging availability of NGS-based MGPT allowed the expansion of patients eligible for germline testing, however, the missing heritability in LS can only be investigated by the concurrent analysis of both the coding and non-coding regions^4^. Our diagnostic center has previously confirmed the robustness of WGS in detecting noncoding PVs in families with familial adenomatous polyposis and the Carney complex^6,24^. However, the optimal place of WGS in the molecular genetic diagnostic workflow of LS has not been determined.

Therefore, we designed a straightforward diagnostic algorithm for LS. Following national and international guidelines^8^, the first step is the application of the NGS-based MGPT. If this test fails to detect a PV, the lack of somatic *MLH1* promoter methylation in MLH1-deficient tumors suffices the need for WGS. Although there are examples of rendering patients with somatic *MLH1* promoter methylation ineligible for LS germline testing^4,25^, the instances of LS-associated cancers with *MLH1* promoter methylation^25^ convinced us not to exclude these samples from further analysis. Subsequent evaluation of samples utilized RNA-based identification of a gDNA-directed transcriptional alteration, a method which has previously been widely applied^14,26,27^. In particular, we tested if an allelic imbalance of a germline transcribed marker detected by the MGPT might provide evidence of NMD caused by a frameshift PV^28^, in which case WGS would also be advised. This prioritization algorithm can be safely regarded as a time- and cost-efficient workflow. We have recently demonstrated that the application of a pan-cancer hereditary MGPT to diagnose hereditary endocrine tumor syndromes is a cost-effective strategy^29^. Following MGPT, the cost of performing both the *MLH1* promoter methylation analysis and the cDNA-based investigation of allelic imbalance is approximately 300 USD, while WGS followed by clinical variant annotation and confirmatory validation by an independent method is available between 3000 and 4000 USD^30,31^. Although inititatives to reduce sequencing costs by performing low-pass WGS might allow the detection of CNVs^32,33^, current diagnostic procedures necessitate 30-50X average read depth to allow adequate SNV calling.

During the 19-month prospective implementation period of this workflow, MGPT has identified 31 PVs (31%) in LS genes, which correlates well with results from different centers^34–36^. Among these PVs, 3 (9.7%) were complex variants. Molecular characterization of these complex PVs revealed the transcriptional consequences, while all of them were confirmed to be recurrent in our studied population. The two novel SVs have not been previously identified, however in the case of *MSH2* c.[2042 A > C; 2045 C > T], c.2045 C > T has been previously detected in another patient with a clear germline MSH2 PV (c.1292 T > A), supporting the neutral effect of c.2045 C > T^37^. This is important as only c.2042 A > C can be regarded as a PV, which presumably first appeared on a haplotype containing c.2045 C > T as no occurrence of c.2042 A > C without c.2045 C > T has been observed.

The WGS-based detection of an *MLH1* deep intronic PV resulting in a novel splice donor and causing the exonization of a 288bp-long intronic region in the only sample selected for WGS from our prospective cohort clearly shows the benefit of adding WGS to the diagnostic procedure. Interestingly, this very variant was also detected in our retrospective cohort in a patient with MLH1-deficient CRC and a clear familial history of LS-associated cancers, while it was absent in the gnomAD database (gnomAD Genomes 3.1.2 v2) reporting the results of 75,000 human genomes.

The recurrent nature of all the identified complex PVs as well as the detected *MLH1* deep intronic PV greatly highlights the clinical importance of this workflow in the studied population. Our method also confirms the necessity of a multimodal approach in the genetic diagnostic workflow of LS, including genetic counseling, DNA and RNA-based genetic testing and expert histopathological diagnosis. This enables optimal variant classification and is a prerequisite to performing proper presymptomatic genetic testing in relatives, who can then benefit from personalized cancer screening protocols.

It is important to note the following limitations of this study. Primarily, although this is the first prospective analysis of the role of WGS in the diagnosis of LS, our 19-month timeframe allowed the inclusion of only 100 patients. Next, our algorithm is unable to leverage allelic imbalance assays in samples with no germline transcribed heterozygote markers and therefore, might still miss the diagnosis of all deep intronic PVs. Further, there are certain, extra rare cases of hereditary epigenetic variations resulting in Lynch syndrome (e.g. constitutional, primary or secondary methylation of the *MLH1* promoter^38,39^) which might still be missed with this approach. However, as earlier reports confirmed that germline noncoding DNA variations are the most frequent cause of undiagnosed LS after MGPT^4^, we believe that our approach presents a clinically viable and cost-effective workflow to optimally provide molecular genetic diagnosis of LS. Moreover, the application of the gnomAD database as an universal healthy control population is suboptimal as it does not reflect the germline genetic background of the studied (Hungarian) population, however there is no large germline whole genome database currently available which might have been used in this population. Nevertheless, by the application of the gnomAD database we were able to filter out variants which might have been found to be frequent in only one, distinct population, which can help to robustly filter out benign variants^40^.

In conclusion, we designed a biomarker-driven prioritization workflow for the incorporation of WGS in the diagnosis of LS. By implementing this workflow on 100 consecutive patients diagnosed at our center, 3 complex PVs were identified out of 31 PVs with MGPT and 1 deep-intronic PV has been detected with WGS. Further analysis confirmed the recurrent nature of all these complex and deep-intronic PVs which underlines the clinical importance of this workflow in our studied population.

## Supplementary information

Supplementary Information

Supplementary Data

## Data Availability

All data generated or analyzed during this study are included in this published article [and its supplementary information files]. Previously unreported variants have been deposited in the Leiden Open Variation Database (LOVD, variant IDs: 0000959750, 0000959748, 0000959747 0000959744, 0000985142, 0000987870).
