# Supplementary material for: Whole genome sequencing completes the molecular genetic testing workflow of patients with Lynch syndrome
Source: NPJ Genom Med. 2025 Jan 18;10:5. doi: 10.1038/s41525-025-00461-z (PMC11742971; doi:10.1038/s41525-025-00461-z)
Supplement: Supplementary file 1 — Supplementary Information [file 41525_2025_461_MOESM1_ESM.pdf]

Supplementary Information

Whole genome sequencing completes the molecular genetic testing workflow of patients with Lynch syndrome

Klaudia Horti-Oravecz[1,2,3]*, Anikó Bozsik[1,3,4]*, Tímea Pócza[1], Ildikó Vereczkey[5], Tamás Strausz[5], Erika Tóth[3,5], Tatiana Sedlackova[6,7], Diana Rusnakova[6,7], Tomas Szemes[6,7,8], István Likó[4], Edit Oláh[1], Henriett Butz[1,3,4,9,10], Attila Patócs[1,3,4,9], János Papp[1,3,4#] and Vince Kornél Grolmusz[1,3,4,9#*]

[1] Department of Molecular Genetics, National Institute of Oncology, Budapest, Hungary

[2] Semmelweis University Doctoral School, Budapest, Hungary

[3] National Tumorbiology Laboratory, National Institute of Oncology, Budapest, Hungary

[4] Hereditary Tumors Research Group, HUN-REN – Semmelweis University, Budapest, Hungary

[5] Department of Surgical and Molecular Pathology, National Institute of Oncology, Budapest, Hungary

[6] Comenius University Science Park, Bratislava, Slovakia

[7] Geneton Ltd., Bratislava, Slovakia

[8] Department of Molecular Biology, Faculty of Natural Sciences, Comenius University, Bratislava, Slovakia

[9] Department of Laboratory Medicine, Semmelweis University, Budapest, Hungary

[10] Department of Oncology Biobank, National Institute of Oncology, Budapest, Hungary

* contributed equally

[#] supervised jointly

[*] correspondence: grolmusz.vince@oncol.hu

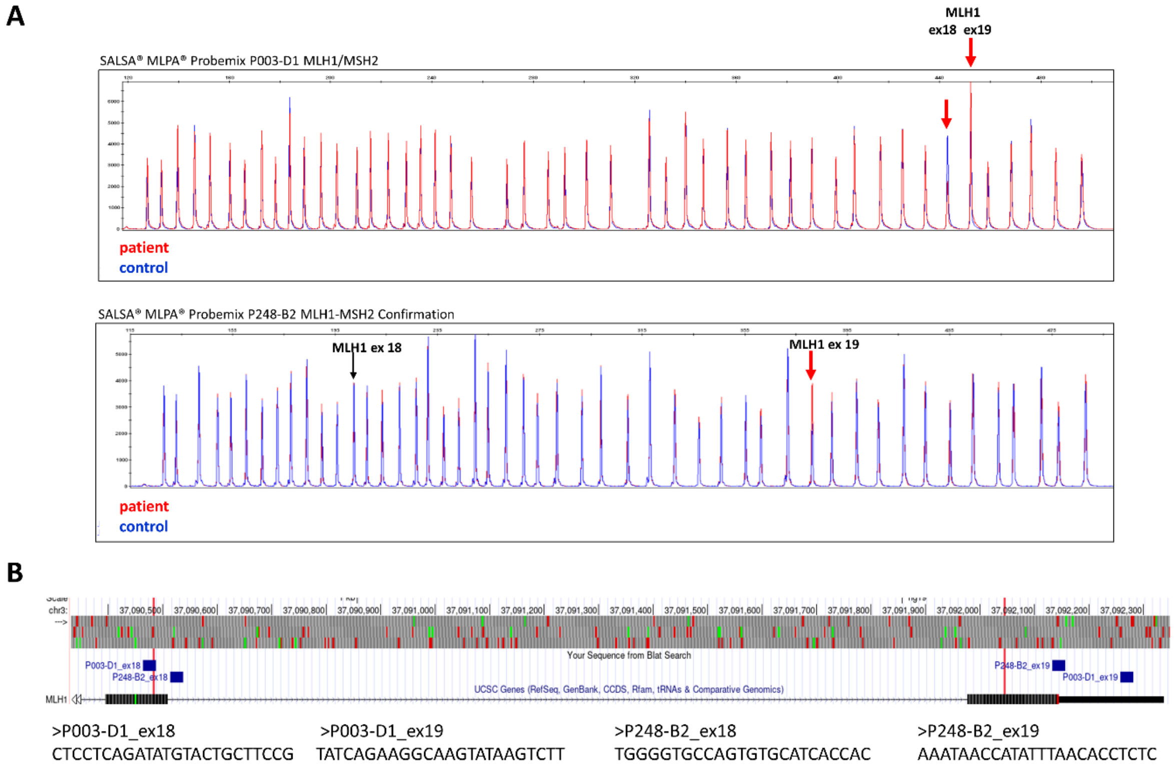

Figure S1 – MLPA and confirmation MLPA analysis of a novel SV in *MLH1* Visual representation of MLPA analysis with the default (P003-D1) and confirmation (P248-B2) probe sets (A). Hybridization of the MLPA probes for MLH1 ex18 and ex19 of MLPA kits P003-D1 MLH1/MSH2 and P248-B2 MLH1-MSH2 Confirmation depicted in UCSC genome browser blat tool (B). Red vertical lines represent the deletion borders of 1563 bp of genomic deletion of *MLH1*. Note: In P248-B2 MLH1-MSH2 Confirmation probe set the deleted region overlapping ex18 and the inserted region containing ex18 compensated each other in terms of copy number. Contrary, in the P003-D1 MLH1/MSH2 probe set the probe for ex18 concurred with the deletion/duplication breakpoint, therefore it revealed the deletion.

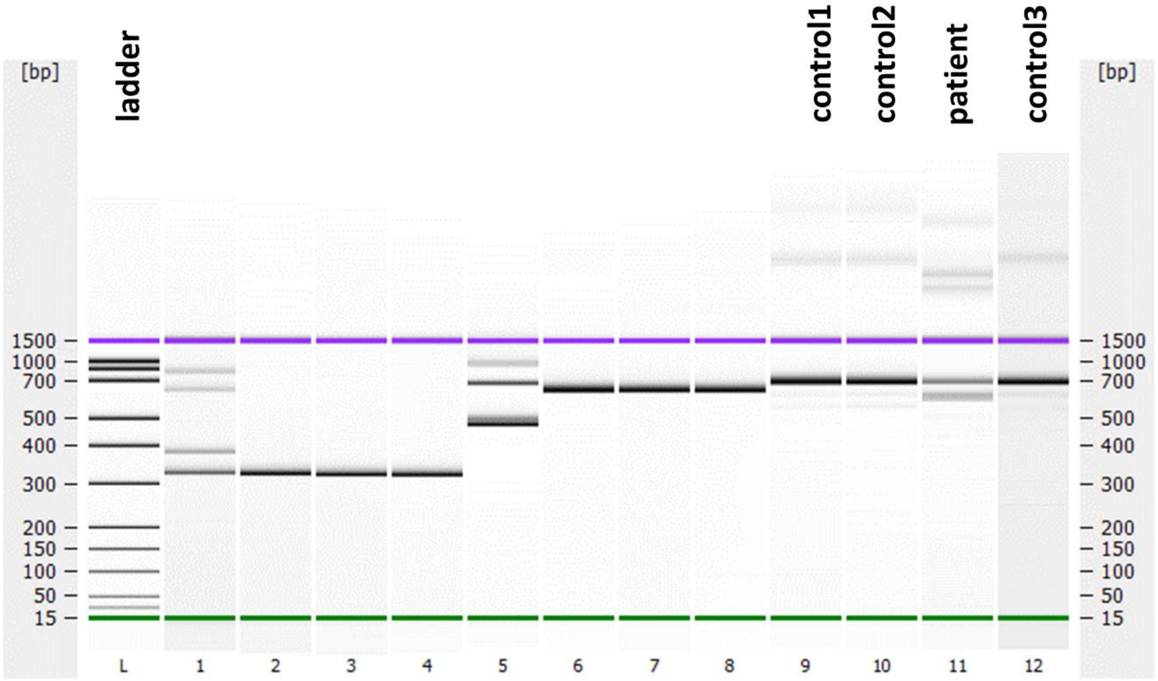

Figure S2 – Uncropped scan of electrophoretic separation image related to Figure 2D. Note: Lanes between the ladder and control 1 include samples from unrelated projects.

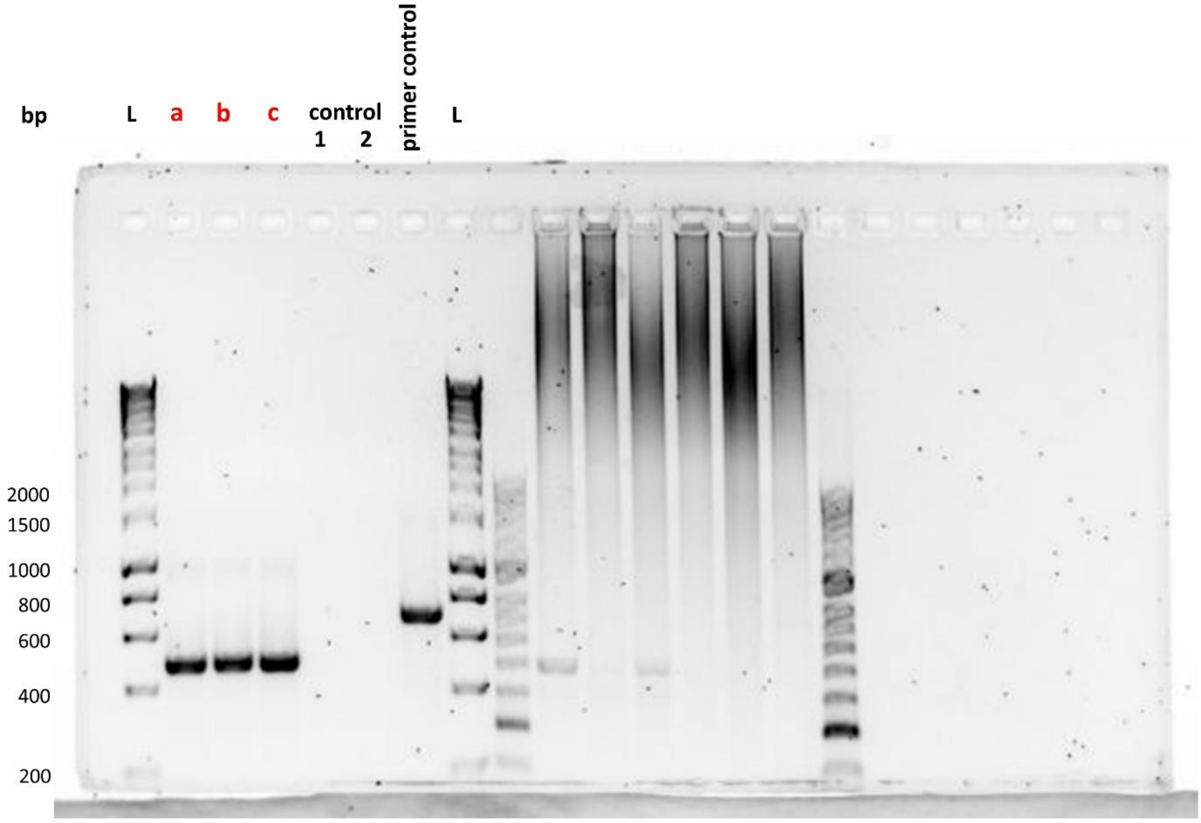

Figure S3 – Uncropped scan of gel blot related to Figure 2G. Note: Unannotated lanes include samples from unrelated projects.

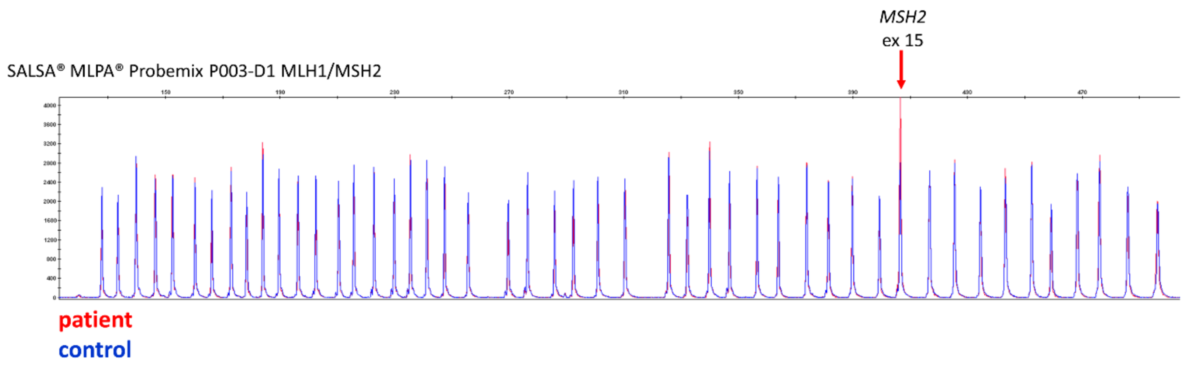

Figure S4 – MLPA analysis of a novel SV in *MSH2* (*MSH2* c.2620_2621ins[G;2507_2620]).Visual representation of MLPA analysis (probeset #P003-D1).

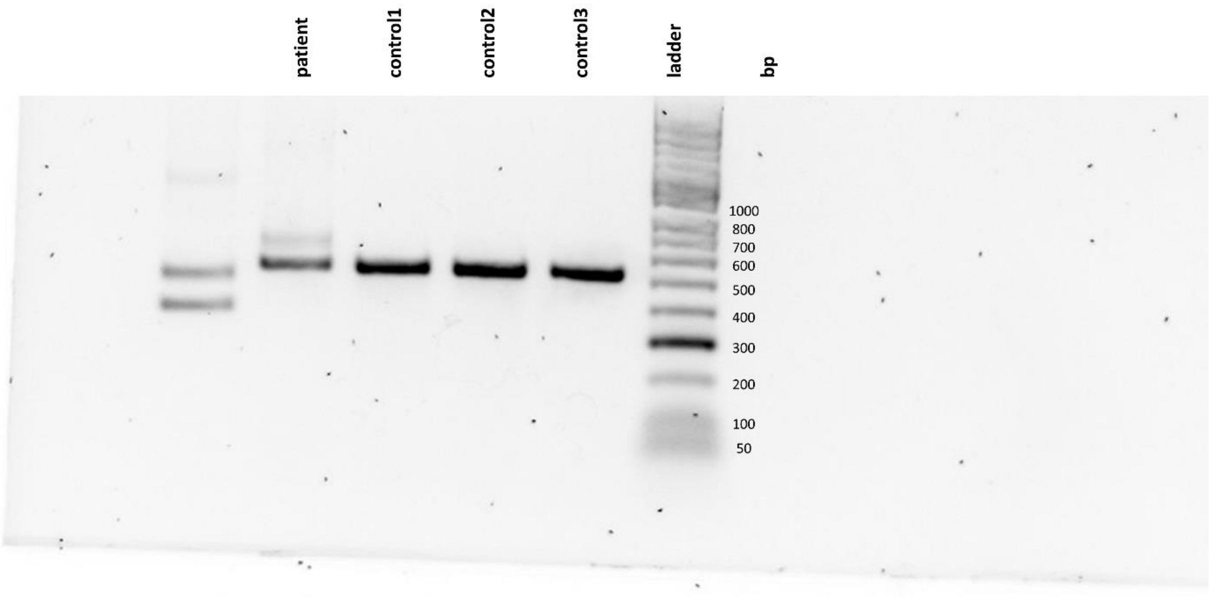

Figure S5 – Uncropped scan of gel blot related to Figure 3D. Note: Unannotated lane include sample from an unrelated project.

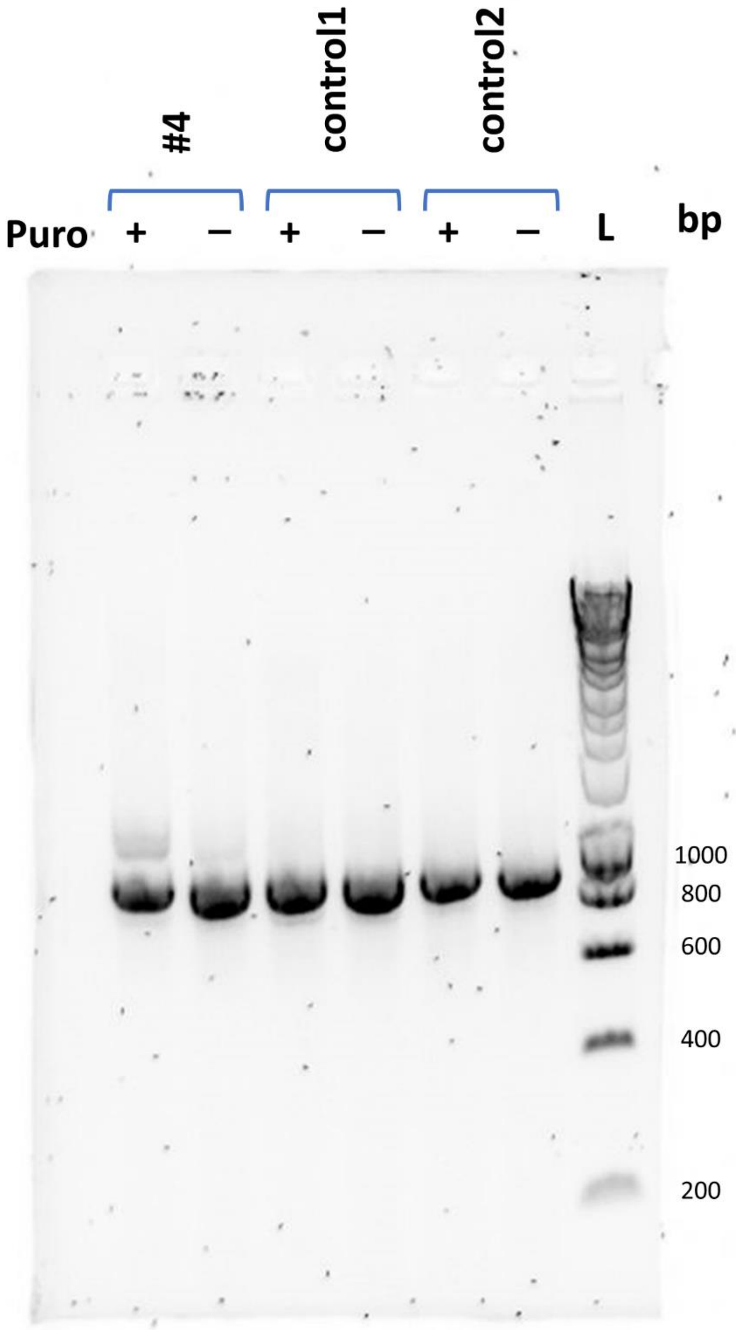

Figure S6 – Uncropped scan of gel blot related to Figure 5C.

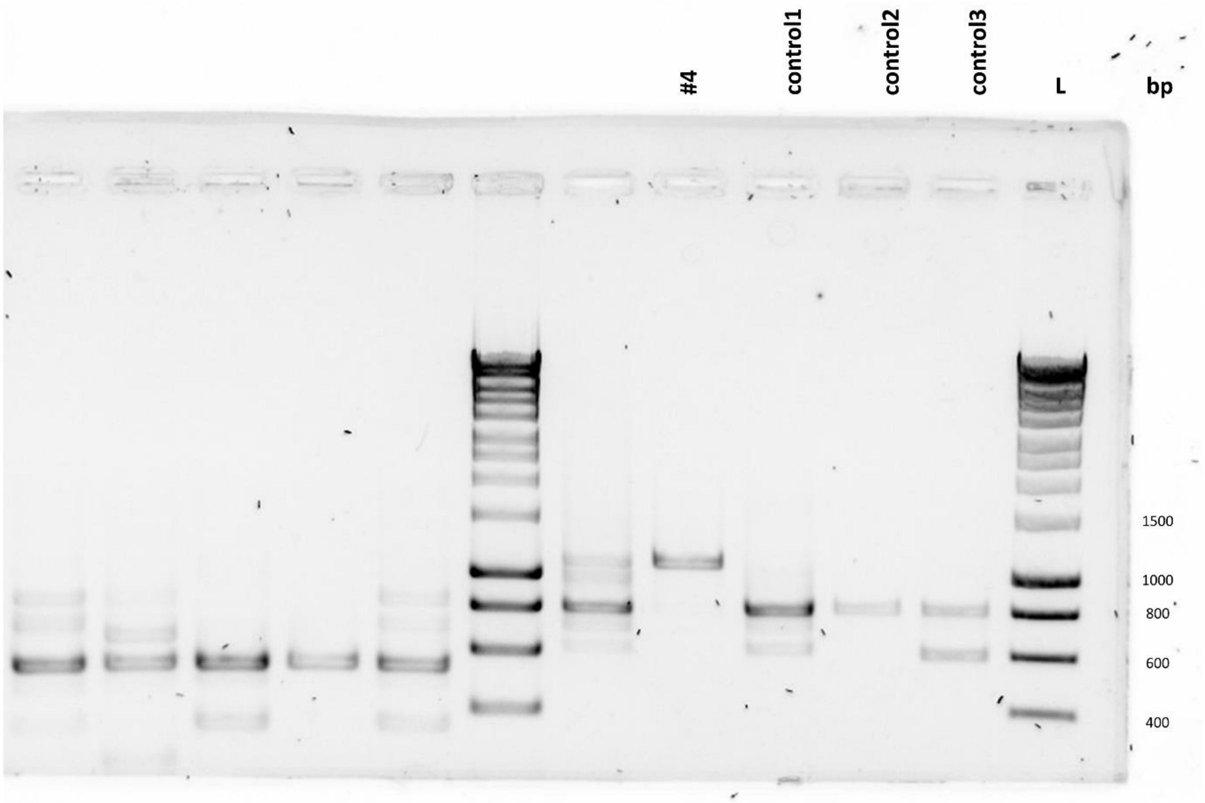

Figure S7 – Uncropped scan of gel blot related to Figure 5D. Note: Unannotated lanes include samples from unrelated projects.

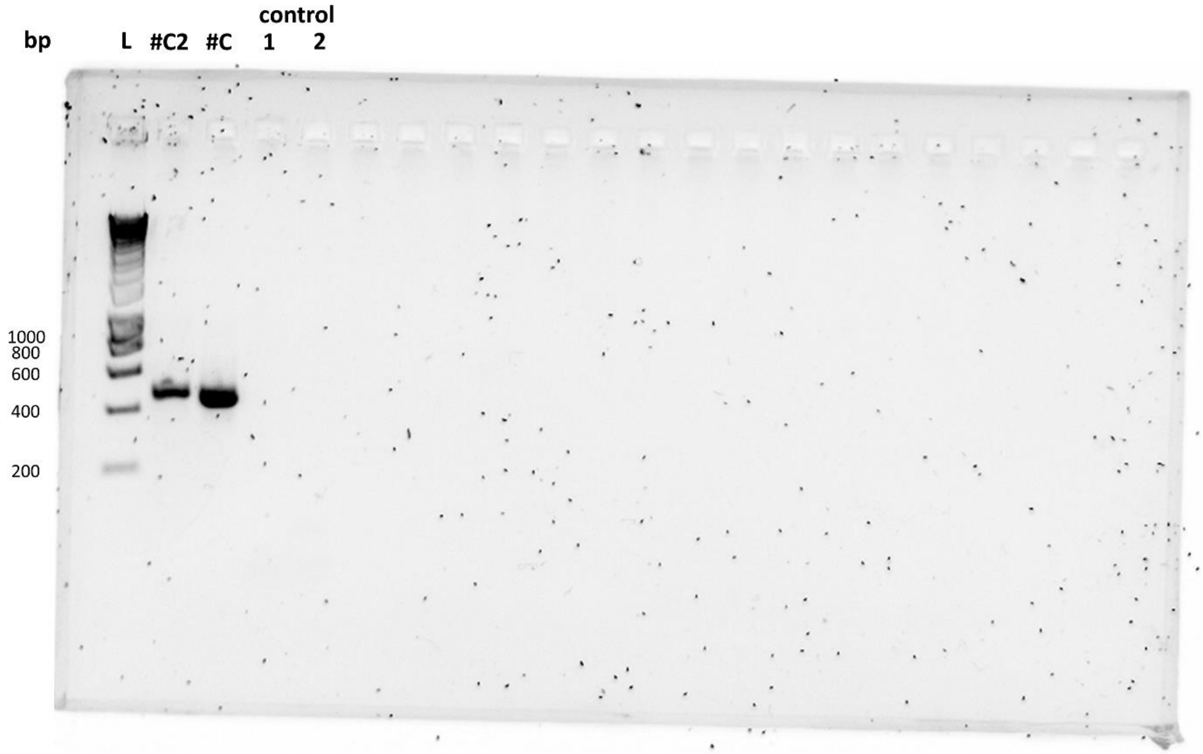

Figure S8 – Uncropped scan of gel blot related to Figure 6G.

Table S1 – Sequences of oligonucleotide primers used in the study – Primers applied for *MLH1* promoter methylation analysis (A). Primers applied for cDNA-based analyses (B). Primers used for the *MLH1* c.[2078_2172del; 2080_*+493dup]-specific PCR (C). Forward cDNA primer for the detection of *MSH2* c.2620_2621ins[G;2507_2620] (D). PCR primers used in the characterization of *MLH1* c.306+1222A>G (E).

A

MLH1 promoter primers

MLH1-Bis_for	5'-TTTTTAGGAGTGAAGGAGG-3'
MLH1-Bis_rev	5'-ATAAAACCCTATACCTAATCTATC-3'

B

cDNA primers

gene	amplicon	primer ID	primer sequence
MLH1	**A**	MLH1_C1-F	5'-CGTTGAGCATCTAGACGTTTCC-3'
		MLH1_C2-R	5'-CCAGACGATGGTTGATGAAGA-3'
	**B**	MLH1_C2-F	5'-ACCTATGGCTTTCGAGGTGAG-3'
		MLH1_C3-R	5'-CGAGGTCAGACTTGTTGTGGA-3'
	**C**	MLH1_C3-F	5'-TGAGGATAAAACCCTAGCCTTCA-3'
		MLH1_C4-R	5'-CCAGAAAGAGACATCGGGAAG-3'
	**D**	MLH1_C5-F	5'-CTTGGAGGGGGATACAACAAA-3'
		MLH1_C6-R	5'-ACTGCTTCCGGATGGAATAGA-3'
	**E**	MLH1_C6-F	5'-CTTCGTGGGCTGTGTGAATC-3'
		MLH1_C7-R	5'-ATCCCACAGTGCATAAATAACCATA-3'
MSH2	**A**	MSH2_C1-F	5'-GCATTTTCTTCAACCAGGAGGT-3'
		MSH2_C2-R	5'-ACCGGTTGAGGTCCTGATAAA-3'
	**B**	MSH2_C2-F	5'-GCTGGAAATAAGGCATCCAAG-3'
		MSH2_C3-R	5'-CTGCCTCAATTCTGCATCTTCT-3'
	**C**	MSH2_C3-F	5'-GCTGGAGACATGGGGAAACT-3'
		MSH2_C4-R	5'-CAAGGAATTCATGGTTTTCCAC-3'
	**D**	MSH2_C4-F	5'-CCCCTCAAGGACAAAGACTTG-3'
		MSH2_C5-R	5'-GGAACAGGTGCTCCATTTGAC-3'

	**E**	MSH2_C5-F	5'-TCCTCTTACTGATCTTCGTTCTGACT-3'
		MSH2_C6-R	5'-TAGCCCATGCTAACCCAAATC-3'
	**F**	MSH2_C6-F	5'-GCTCAGCTAGATGCTGTTGTCA-3'
		MSH2_C7-R	5'-GCATTTGTTTCACCTTGGACA-3'
MSH6	**A**	MSH6_C1-F	5'-AGATGCGGTGCTTTTAGGAG-3'
		MSH6_C2-R	5'-AGGGGTAACCCTCCATCTTG-3'
	**B**	MSH6_C2-F	5'-AGATCGGTAGCGCCTGCTGC-3'
		MSH6_C3-R	5'-TCACTGCTTCCTTCCTCCTT-3'
	**C**	MSH6_C3-F	5'-CCTCAGAGCCAGAAGAGGAA-3'
		MSH6_C4-R	5'-TCTACATCGTGCCTCCATCA-3'
	**D**	MSH6_C4-F	5'-GTGCCCCACTCTGTAACCAT-3'
		MSH6_C5-R	5'-ACCATCACCCCCTCGACTAT-3'
	**E**	MSH6_C5-F	5'-GCTGAAGAACGGAGGGATGT-3'
		MSH6_C6-R	5'-CATTCTCTGGTGCTTGTGGA-3'
	**F**	MSH6_C6-F	5'-CCCAGATGGGTTGTTACGTC-3'
		MSH6_C7-R	5'-CAATTTATGGACAGCTTCAGCA-3'
PMS2	**A**	PMS2_cDNS_01_F	5'-CTGGAGGGAACTTTCCCAGT-3'
		PMS2_cDNS_01_R	5'-CTTCCACCTGTGCATACCAC-3'
	**B**	PMS2_cDNS_02_F	5'-CTGTGCGCCATAAGGAATTT-3'
		PMS2_cDNS_02_R	5'-AGGGGATTGATCCTGCTTTT-3'
	**C**	PMS2_cDNS_03_F	5'-TGTCAACAAGCTAAATGTCAGTCA-3'
		PMS2_cDNS_03_R	5'-CCAGGGGCACAACTTTCTTA-3'
	**D**	PMS2_cDNS_04_F	5'-TCAGGTTGATGTAGCTGTGAAAA-3'
		PMS2_cDNS_04_R	5'-AGAAATGACACCCAGGTTGG-3'

C

gDNA primers for the detection of *MLH1* c.[2078_2172del; 2080_*+493dup]

MLH1_int18_tp_AS	5'-CTCCTAAAGATTGTATGAGGTCCTGTC-3'
MLH1_int18_tp_S	5'-AGCACTTAAGACTTATACTTGCCTTC-3'

D

forward cDNA primer for the detection of *MSH2* c.2620_2621ins[G;2507_2620]

MSH2_C7-F	5'-ACGTTCATGGCTGAAATGTTG-3'

E

PCR primers used in the characterization of *MLH1* c.306+1222A>G

MLH1_promoter_cDNS_F	5'-GAGACCCAGCAACCCACAG-3'
MLH1_promoter_cDNS_R	5'-TCATCTCTTTGATAGCATTAGCTG-3'
MLH1_int03_F	5'-TGGGTAGAGACAGGGTTTGG-3'
MLH1_int03_R	5'-TTCTTTTACCAAGGAATGTTATTTC-3'
MLH1_c.-93G_allele_spec_F	5'-GATGGCGTAAGCTACAGCTG-3'
MLH1_C1-R	5'-GGGCTTTCAGTTTTCCATCTG-3'
MLH1_ex04-ex03_R	5'-TATGCTGGCCAAAGCCTCA-3'

Table S2 – DRAGEN SV analysis in patient #10 – Report output of the DRAGEN SV analysis concerning the detected SV in *MLH1* in patient #10.

#CHROM	POS	ID	REF	ALT	QUAL	FILTER	INFO	FORMAT	sample
chr3	37090480	MantaDEL:128:0:1:4:0:0	C	<DEL>	999	PASS	END=37092043;SVTYPE=DEL;SVLEN=-1563;CIPOS=0,2;CIEND=0,2;HOMLEN=2;HOMSEQ=TG	GT:FT:GQ:PL:PR:SR	0/1:PASS:999:999,0,999:108,18:245,82
chr3	37090483	MantaBND:128:0:1:8:0:0:0	A	]chr3:37092635]A	545	PASS	SVTYPE=BND;MATEID=MantaBND:128:0:1:8:0:0:1;CIPOS=0,2;HOMLEN=2;HOMSEQ=GG;BND_DEPTH=229;MATE_BND_DEPTH=22	GT:FT:GQ:PL:PR:SR	0/1:PASS:545:595,0,999:31,5:69,17
chr3	37092635	MantaBND:128:0:1:8:0:0:1	A	A[chr3:37090483[	545	PASS	SVTYPE=BND;MATEID=MantaBND:128:0:1:8:0:0:0;CIPOS=0,2;HOMLEN=2;HOMSEQ=AG;BND_DEPTH=22;MATE_BND_DEPTH=229	GT:FT:GQ:PL:PR:SR	0/1:PASS:545:595,0,999:31,5:69,17

Table S3 – **DRAGEN SV analysis in patients #C and #D** – Report output of the DRAGEN SV analysis concerning the detected SV in *MLH1* in patients #C and #D.

patient ID	#CHROM	POS	ID	REF	ALT	QUAL	FILTER	INFO	FORMAT	sample
C	chr3	37090480	DRAGEN:DEL:976:0:3:0:0:0	C	<DEL>	999	PASS	END=37092043;SVTYPE=DEL;SVLEN=-1563;CIPOS=0,2;CIEND=0,2;HOMLEN=2;HOMSEQ=TG	GT:FT:GQ:PL:PR:SR	0/1:PASS:999:999,0,999:161,9:397,116
C	chr3	37090483	DRAGEN:BND:976:0:2:0:0:0:1	A	]chr3:37092635]A	678	PASS	SVTYPE=BND;MATEID=DRAGEN:BND:976:0:2:0:0:0:0;CIPOS=0,2;HOMLEN=2;HOMSEQ=GG;BND_DEPTH=295;MATE_BND_DEPTH=35	GT:FT:GQ:PL:PR:SR	0/1:PASS:678:728,0,999:43,2:113,23
C	chr3	37092635	DRAGEN:BND:976:0:2:0:0:0:0	A	A[chr3:37090483[	678	PASS	SVTYPE=BND;MATEID=DRAGEN:BND:976:0:2:0:0:0:1;CIPOS=0,2;HOMLEN=2;HOMSEQ=AG;BND_DEPTH=35;MATE_BND_DEPTH=295	GT:FT:GQ:PL:PR:SR	0/1:PASS:678:728,0,999:43,2:113,23
D	chr3	37090480	DRAGEN:DEL:362:0:1:0:0:0	C	<DEL>	999	PASS	END=37092043;SVTYPE=DEL;SVLEN=-1563;CIPOS=0,2;CIEND=0,2;HOMLEN=2;HOMSEQ=TG	GT:FT:GQ:PL:PR:SR	0/1:PASS:999:999,0,999:186,25:361,110
D	chr3	37090483	DRAGEN:BND:362:0:2:0:0:0:0	A	]chr3:37092635]A	608	PASS	SVTYPE=BND;MATEID=DRAGEN:BND:362:0:2:0:0:0:1;CIPOS=0,2;HOMLEN=2;HOMSEQ=GG;BND_DEPTH=283;MATE_BND_DEPTH=27	GT:FT:GQ:PL:PR:SR	0/1:PASS:608:658,0,999:44,2:109,21
D	chr3	37092635	DRAGEN:BND:362:0:2:0:0:0:1	A	A[chr3:37090483[	608	PASS	SVTYPE=BND;MATEID=DRAGEN:BND:362:0:2:0:0:0:0;CIPOS=0,2;HOMLEN=2;HOMSEQ=AG;BND_DEPTH=27;MATE_BND_DEPTH=283	GT:FT:GQ:PL:PR:SR	0/1:PASS:608:658,0,999:44,2:109,21

Table S4 – Clinical characteristics of the retrospective cohort selected for WGS analysis
Abbreviations: M – male, F – female, CRC – colorectal cancer, DC – duodenal cancer, EC – endometrial cancer, PanC – pancreatic cancer, GC – gastric cancer, BrainT – brain tumor, KC – kidney cancer, MSI-H – high levels of microsatellite instability.

patient ID	sex	age (years)	personal cancer history	familial (LS-associated) cancer history
W1	F	59	CRC50	sister CRC52, mother EC55
W2	F	27	CRC25	father CRC45, paternal grandmother CRC50
W3	M	57	CRC56	mother CRC50, maternal grandmother CRC57
W4	F	35	CRC34	paternal aunt CRC58, paternal grandfather GC69
W5	M	38	CRC37	daughter BrainT2
W6	F	38	CRC38	sister CRC32
W7	M	56	CRC47	father KC75, mother KC82, maternal aunt PanC
W8	F	43	CRC42(MSI-H)	brother CRC35, maternal aunt CRC64, paternal grandfather CRC74
W9	M	56	DC(MSI-H)	mother PanC52
W10	M	60	CRC52, CRC59(MSI-H)	mother KC49, sister CRC49, maternal uncle CRC43, maternal grandmother GC52

Legends for Suppementary Data

Supplementary Data S1 – *MLH1* SNVs detected in patient #4 with WGS

Supplementary Data S2 – Rare SNVs in *MLH1, MSH2, MSH6, PMS2* and *EPCAM* detected in patients #W1-10 with WGS Variants were filtered as described in Figure 7.
